# Urbanisation drives parallel genomic divergence across cities and bumble bee species

**DOI:** 10.1186/s13059-026-04322-5

**Published:** 2026-10-26

**Authors:** Panagiotis Theodorou, Robert J. Paxton

**Affiliations:** 1 General Zoology, Institute for Biology, Martin Luther University Halle-Wittenberg, Hoher Weg 8, Halle (Saale), 06120 Germany; 2 German Centre for Integrative Biodiversity Research (iDiv) Halle-Jena-Leipzig, Puschstrasse 4, Leipzig, 04103 Germany

**Keywords:** Genotype–environment association, Urban evolution, Landscape genomics, Selective sweeps, Population genomics, Whole-genome sequencing

## Abstract

**Background:**

Urbanisation imposes pervasive environmental change, yet it remains unclear whether evolutionary responses to cities are largely idiosyncratic or whether similar selective pressures across cities repeatedly generate predictable genomic outcomes.

**Results:**

Here, we analyse whole-genome data from 524 individuals of three widespread bumble bee species sampled across nine independent urban–rural pairs in Central Europe to test whether urban environments drive parallel evolutionary responses across cities and species. Genetic diversity responded idiosyncratically to urbanisation, with two species showing no detectable urban–rural differences and one species exhibiting reduced diversity in cities, consistent with species-specific ecological traits and habitat requirements. Despite weak population structure and high gene flow in all species, we detected consistent and replicated genomic divergence associated with urban environments. Genome–environment association analyses revealed parallel allele-frequency shifts at the same loci across independent cities for each species, while selective sweep scans identified repeated signals of positive selection in urban populations. Across species, urban-associated divergence was highly polygenic and occurred predominantly in non-coding regions with potential regulatory roles. Although the specific genes involved were largely species-specific, functional enrichment analyses revealed strong convergence across species, particularly in pathways related to physiological stress responses, neurocognitive and behavioural processes, metabolism and immunity.

**Conclusions:**

Together, these results suggest that urban environments can impose recurrent selective pressures, generating predictable and parallel genomic responses across cities and species despite ongoing gene flow. More broadly, our findings highlight polygenic, potentially regulatory adaptation as an important component of evolutionary responses to urban environments.

**Supplementary Information:**

The online version contains supplementary material available at https://doi.org/10.1186/s13059-026-04322-5.

## Background

Urban environments represent one of the most extensive and rapidly expanding forms of anthropogenic environmental change, reshaping landscapes worldwide and exposing organisms to novel and intense selective pressures [1–3]. More than half of the world’s human population now lives in cities, a proportion projected to increase to 68% by 2050, accompanied by an expansion of 0.6–1.3 million km^2^ in global urban land cover [4, 5]. Urban development increases impervious surfaces, fragments and degrades habitats, and intensifies multiple stressors, including air, water, light and noise pollution [6]. Together, these changes create a suite of recurrent environmental challenges that are broadly comparable across cities [7], making urban environments powerful, replicated natural experiments for studying evolutionary responses to anthropogenic change [1, 2, 7, 8]. Determining how populations respond to these replicated selective regimes is therefore central to assessing the predictability of evolution and the capacity of species to persist in increasingly urbanised landscapes.

Consistent with this view, a growing body of research demonstrates that urban environments influence fitness-related traits, physiology, behaviour and life-history strategies across diverse taxa [2, 9–11]. Urban-associated phenotypic shifts have been documented in insects, birds, mammals, and plants, affecting traits such as phenology, thermal tolerance, body size, dispersal behaviour, and resource use [2, 7, 11–19]. These findings illustrate how urban environments can drive rapid and often repeatable phenotypic change. However, while phenotypic responses are increasingly well characterised, comparatively few studies have examined their genetic basis at the genome-wide scale across replicated urban systems [17, 20–29]. This limits our ability to determine whether observed phenotypic shifts reflect predictable evolutionary responses to urbanisation and whether similar selective pressures consistently shape genomic variation across taxa and regions.

Recent population genomic studies have begun to address this gap, but many remain constrained by limited genomic resolution, small numbers of populations or a lack of independent replication across cities [2]. As a result, it remains unclear whether urban-associated genetic divergence primarily reflects idiosyncratic, species-specific responses or whether urban environments impose sufficiently consistent selective pressures to generate parallel genomic signatures across populations and species [8]. Resolving this uncertainty is central to understanding the predictability of evolution under rapid anthropogenic change [30] and to distinguishing adaptive responses from non-adaptive processes [31]. Moreover, the genetic architecture underlying such responses remains poorly understood. Theory and empirical studies of adaptation to complex and heterogeneous environments predict that evolutionary responses are often highly polygenic [26, 27, 32–34] and frequently involve regulatory variation rather than large-effect coding mutations [26, 35, 36]. Under this framework, adaptation to urban environments may therefore involve coordinated allele-frequency shifts across many loci, including variants in non-coding regulatory regions.

Directly evaluating these predictions requires comparative population genomic approaches that combine genome-wide resolution with replication across independent urban–rural contrasts and multiple syntopic species [7, 11]. Such designs provide a powerful framework for assessing whether genetic diversity responds consistently to urbanisation and whether allele-frequency shifts are repeated across independent populations. They further enable tests of whether urban-associated divergence reflects polygenic genetic architectures involving coordinated shifts across many loci—including variants in non-coding regulatory regions—and whether similar functional pathways are repeatedly implicated across species. In species characterised by large effective population sizes and high dispersal, detecting parallel genomic divergence under such designs would provide strong evidence for selection acting on standing genetic variation and for divergence emerging in the face of gene flow.

Insects are particularly well suited to address these questions because of their short generation times, large population sizes and strong ecological interactions [37, 38]. Among insects, bees represent an informative model system: they occur across urban and rural landscapes, experience multiple urban-associated stressors, and show substantial interspecific variation in ecology, life-history traits and resource use [39, 40]. Many bee species also maintain high levels of gene flow, providing an opportunity to test whether urban-associated genetic divergence can emerge despite weak population structure [27, 41–44]. Urban ecological studies have documented pronounced effects of urban environments on bee fitness, population dynamics and community composition, with outcomes strongly dependent on species-specific habitat requirements, dispersal abilities and life-history traits [45–56]. However, far less is known about how these ecological effects translate into evolutionary and genomic responses in urban bee populations (cf. [27, 44, 57–60]). Consequently, it remains unclear whether genomic responses to urban environments are consistent and predictable across cities and species or dominated by idiosyncratic dynamics.

Here, we use a replicated comparative population genomic framework to investigate evolutionary responses to urban environments in three widespread, syntopic bumble bee species (*Bombus lapidarius*, *Bombus terrestris* and *Bombus pascuorum*) sampled across nine independent pairs of European cities and rural landscapes. The three species differ in key ecological traits, including body size, nesting biology and floral resource use, providing a comparative framework for testing whether genomic responses to urbanisation are shared across species or instead shaped by species-specific ecologies. In particular, *B. pascuorum* is a smaller, long-tongued species that typically visits long-corolla flowers and nests above ground, or close to the soil surface in dense vegetation, whereas *B. lapidarius* and *B. terrestris* are larger, shorter-tongued and more generalist flower visitors that typically nest in subterranean cavities [61]. Using whole-genome sequence data from 524 individuals, we quantify genome-wide genetic diversity, assess genome-wide patterns of divergence associated with urban environments, and test for signatures of selection shared across cities and species. Specifically, we combine genome–environment association analyses with selective sweep scans and functional annotation to identify loci and biological pathways potentially involved in responses to urban living. Based on evolutionary theory and prior empirical work [2, 8, 11, 27, 44], we test four hypotheses: (1) urban environments affect genome-wide genetic diversity in a species-dependent manner, reflecting species-specific ecological traits rather than uniform reductions in urban populations; (2) urban environments drive consistent intraspecific allele-frequency shifts across replicated cities, such that urban populations exhibit parallel genetic divergence from rural populations despite high gene flow; (3) genomic responses to urban environments are predominantly polygenic and enriched in non-coding regions with potential regulatory roles; and (4) convergence in urban-associated genomic responses occurs across species, particularly at the level of gene function, involving pathways related to physiological stress responses, neural and behavioural processes, metabolism and immunity. Together, these hypotheses allow us to assess whether urban environments generate predictable evolutionary outcomes across species and cities and to identify convergent genomic signatures associated with one of the most widespread environmental changes of the Anthropocene.

## Results and discussion

### Genetic diversity, divergence and population structure across urban and rural landscapes

**Fig. 1:**
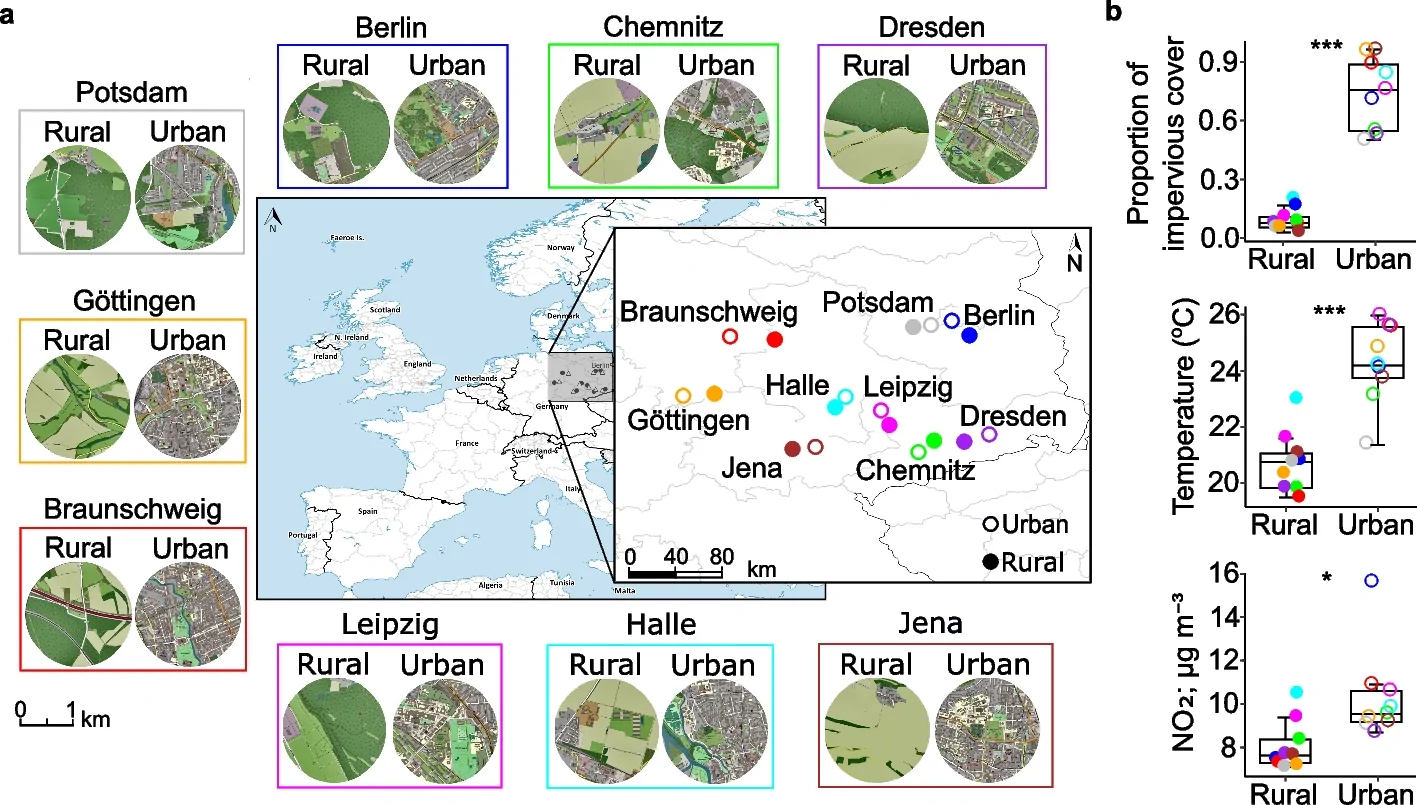
Sample sites and intensity of urbanisation. **a** Locations of paired urban and rural sampling sites across Germany. Cities are labelled; open circles denote urban sites and filled circles denote rural sites, with paired sites shown in matching colours. Land-use categories are indicated as follows: agricultural areas (light green), semi-natural, forest and urban green areas (dark green), and residential areas and buildings (grey). Maps were created using Natural Earth vector and raster data (naturalearthdata.com) and QGIS Desktop. Natural Earth data are licensed under the Creative Commons Attribution–ShareAlike 3.0 licence (CC BY-SA; https://creativecommons.org/licenses/by-sa/3.0/). Additional spatial data were provided by OpenStreetMap contributors and licensed by the OpenStreetMap Foundation under the Open Database License (ODbL; https://opendatacommons.org/licenses/odbl/). **b** Box plots (medians and interquartile ranges) showing the proportion of impervious surface cover (roads and buildings), land-surface temperature (°C), and surface concentrations of nitrogen dioxide (NO_2_; µg m⁻^3^) within a 1-km radius of each sampling site, comparing urban and rural locations. Differences between ecosystems were tested using linear mixed-effects models. Asterisks indicate significance levels (*P < 0.05; ***P < 0.001)

After filtering, we retained whole-genome data from 524 individuals across three bumble bee species (*B. lapidarius, B. terrestris* and *B. pascuorum*) sampled from nine paired urban–rural landscapes (Fig. 1a). Final datasets comprised 172 * B. lapidarius* individuals (mean per sampling site = 10, SD = 2; 157,868 SNPs for genetic diversity analyses and 148,057 for population structure), 171 *B. terrestris* individuals (mean = 10, SD = 1; 75,586 and 63,980 SNPs, respectively), and 181 * B. pascuorum* individuals (mean = 10, SD = 1; 528,194 and 497,114 SNPs, respectively).

To ensure that the categorical classification of sampling sites captured consistent environmental contrasts, we compared independent measures of urban intensity and pollution between urban and rural sites using linear mixed‐effects models (LMMs) with site pair included as a random intercept. Urban sampling sites showed significantly higher proportions of impervious surface cover (LMM; *t* = 10.151, *P* < 0.001; Fig. 1b), elevated land-surface temperatures (LMM; *t* = 5.883,* P* < 0.001; Fig. 1b), and increased concentrations of nitrogen dioxide (NO_2_) (LMM; *t* = 2.796, *P* = 0.023; Fig. 1b) and fine particulate matter (PM_2_._5_) (LMM; *t* = 2.736, *P* = 0.025), confirming that the urban–rural pairs represented replicated and environmentally distinct landscapes.

**Fig. 2:**
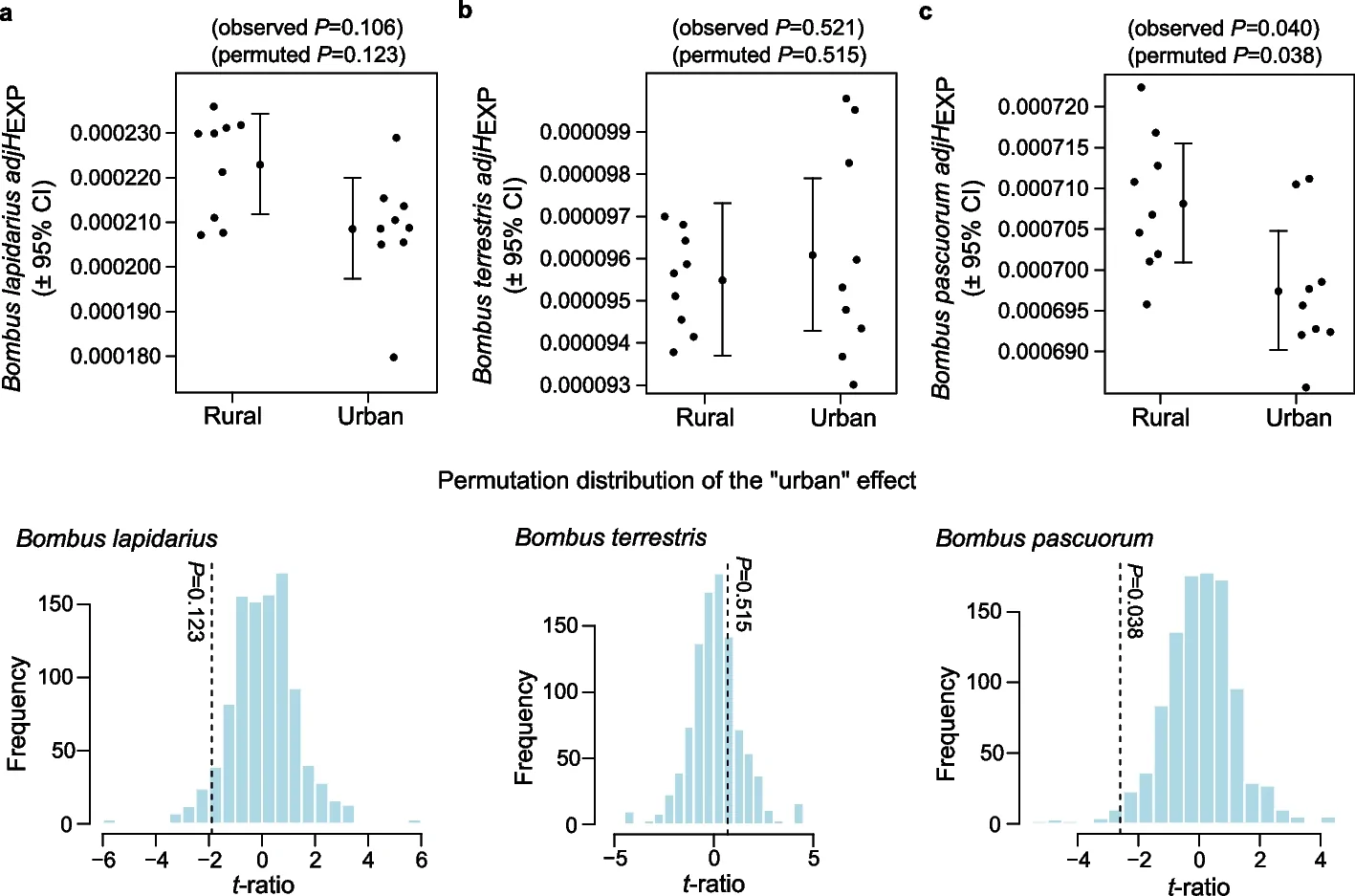
Bumble bee genetic diversity in rural versus urban landscapes. Model-estimated means (± 95% confidence intervals) of adjusted expected heterozygosity (*adjH*_EXP_​) for (**a**) *Bombus lapidarius*, (**b**) *B. terrestris* and (**c**) *B. pascuorum* across rural and urban sampling sites. Points represent partial residuals from linear mixed-effects models with ecosystem type as a fixed effect and site pair as a random intercept, controlling for flowering plant richness and surrounding green cover. Insets show permutation test distributions of the ecosystem effect, with the observed model coefficient indicated by a dashed line

Genome-wide genetic diversity, quantified as adjusted expected heterozygosity using both variant and invariant loci (*adjH*_EXP_; see Methods), ranged from 0.00017–0.00023 in *B. lapidarius*, 0.00009–0.00010 in *B. terrestris*, and 0.00069–0.00072 in *B. pascuorum* (Fig. 2; Additional file 1: Table S1). We used LMMs to test whether genetic diversity differed between urban and rural ecosystems, with site pair included as a random intercept. In addition to ecosystem type, flowering plant richness (estimated using quadrat surveys) and the proportion of green cover within a 1 km radius were included as covariates. In *B. lapidarius*, genetic diversity did not differ significantly between urban and rural sampling sites (LMM; *emmeans*; *t* = –1.899, *P* = 0.106; Fig. 2a) and was not associated with surrounding green cover (LMM; *t* = 1.543, *P* = 0.173) or flowering plant richness (LMM; *t* = 1.967, *P* = 0.096). Similarly, in *B. terrestris*, genetic diversity did not differ between ecosystems (LMM; *emmeans*; *t* = 0.681, *P* = 0.521; Fig. 2b) and was not associated with either flowering plant richness (LMM; *t* = − 1.035, *P* = 0.340) or green cover (LMM; *t* = 1.365, *P* = 0.221). In contrast, *B. pascuorum* exhibited significantly reduced genetic diversity in urban relative to rural sampling sites (LMM; *emmeans*; *t* = –2.598, *P* = 0.040; Fig. 2c). In this species, genetic diversity was also strongly and positively associated with the proportion of green cover surrounding sampling sites (LMM; *t* = 7.663, *P* < 0.001), whereas flowering plant richness showed no detectable effect (LMM; *t* = 0.794, *P* = 0.457). Permutation tests supported these patterns, confirming no significant urban–rural differences in genetic diversity for *B. lapidarius* and *B. terrestris*, but a significant reduction in urban populations of *B. pascuorum* (permuted *P* = 0.038; Fig. 2). Complementary LD-based demographic analyses revealed broadly similar demographic trajectories between ecosystem types in *B. lapidarius* and *B. terrestris* (Additional file 2: Fig. S1). In contrast, urban populations of *B. pascuorum* showed a pronounced decline in the most recent generations, resulting in lower contemporary effective population size estimates relative to rural populations (Additional file 2: Fig. S1).

Genetic differentiation among sampling sites was low in all three species (*B. lapidarius*: mean *F*_ST_ = 0.0016, 95% CI = 0.0011–0.0020; *B. terrestris*: mean *F*_ST_ = 0.0006, 95% CI = 0.0002–0.001; *B. pascuorum*: mean *F*_ST_ = 0.0017, 95% CI = 0.0015–0.0019; Additional file 1: Tables S2–S4), indicating negligible genome-wide differentiation among populations. Consistent with this, Mantel tests revealed no evidence of isolation by distance in any species (*P* > 0.05; Additional file 2: Fig. S2). Multiple complementary analyses of population structure supported these findings: sparse non-negative matrix factorisation (sNMF), *find.clusters*, and discriminant analysis of principal components (DAPC) all identified a single genetic cluster per species (K = 1; Additional file 2: Fig. S3). Together, these results indicate an absence of detectable population structure within each species at the spatial scale of this study.

Urban environments can present both challenges and opportunities for bees [46, 54, 62], and species-specific ecological traits are therefore expected to mediate their demographic and genetic responses to urbanisation. Consistent with this expectation, we observed contrasting effects of urban landscapes on genetic diversity across species. Genetic diversity in *B. lapidarius* and *B. terrestris* did not differ significantly between urban and rural sampling sites and showed no strong associations with local flowering plant richness or surrounding green cover, suggesting that these dietary-generalist, below-ground nesting species are relatively resilient to variation in habitat structure across ecosystems. In contrast, *B. pascuorum* exhibited significantly reduced genetic diversity in urban sampling sites, together with a strong positive association between heterozygosity and surrounding green cover, indicating greater sensitivity to landscape-scale habitat availability.

Reduced genetic diversity in urban *B. pascuorum* populations, together with lower recent effective population size estimates, is consistent with demographic processes that increase the influence of genetic drift, potentially including lower colony densities and increased self-recruitment in urban environments. As a smaller, long-tongued species with greater dietary specialisation than *B. lapidarius* and *B. terrestris* [63, 64], *B. pascuorum* may face particular challenges in fragmented urban landscapes and in locating suitable floral resources. Its above-ground nesting strategy may further increase exposure to elevated temperatures associated with the urban heat island effect [65] and to disturbance from green-space management practices such as mowing and pesticide application [66]. Together, these factors may contribute to reduced population sizes in urban environments. The strong positive association between genetic diversity and green cover further highlights the potential importance of connected green spaces for maintaining viable populations of more specialised bumble bee species.

The near absence of population structure and isolation by distance across all three species is consistent with previous studies of continental bumble bees [27, 39, 67, 68] and reflects the high dispersal capacity of reproductive castes. Bumble bee queens can disperse up to ~ 9 km and males at least 2.6–9.9 km [69, 70], with populations typically occurring at high densities [61, 71–73]. Such conditions are expected to promote panmixia and constrain the emergence of strong genome-wide signals of local adaptation. Importantly, however, they also provide a powerful framework for detecting selection acting on specific loci or genomic regions because consistent allele-frequency shifts across urban populations are unlikely to be explained by population structure or restricted gene flow alone.

### SNPs associated with urban environments

To identify genomic variation associated with urban environments, we combined a constrained ordination approach (redundancy analysis, RDA) with a paired hierarchical linear mixed model (LMM), and used latent factor mixed models (LFMM) as an independent complementary robustness analysis. Redundancy analysis, with ecosystem type (urban versus rural) as the constraining variable, identified 451 urban-associated SNPs in *B. lapidarius* (0.03% of SNPs), 641 in *B. terrestris* (0.03% of SNPs) and 763 in *B. pascuorum* (0.02% of SNPs) (Additional file 1: Tables S5–S7). Across species, RDA explained approximately 6% of total genomic variance, comparable to effect sizes reported in genome–environment association studies of other wild populations [74]. The paired LMM framework (ecosystem type as a fixed effect; site pair as a random effect) detected larger sets of urban-associated SNPs: 4,444 in *B. lapidarius* (0.26% of SNPs), 5,604 in *B. terrestris* (0.25% of SNPs) and 8,271 in *B. pascuorum* (0.26% of SNPs) (Additional file 1: Tables S8–S10).

**Fig. 3:**
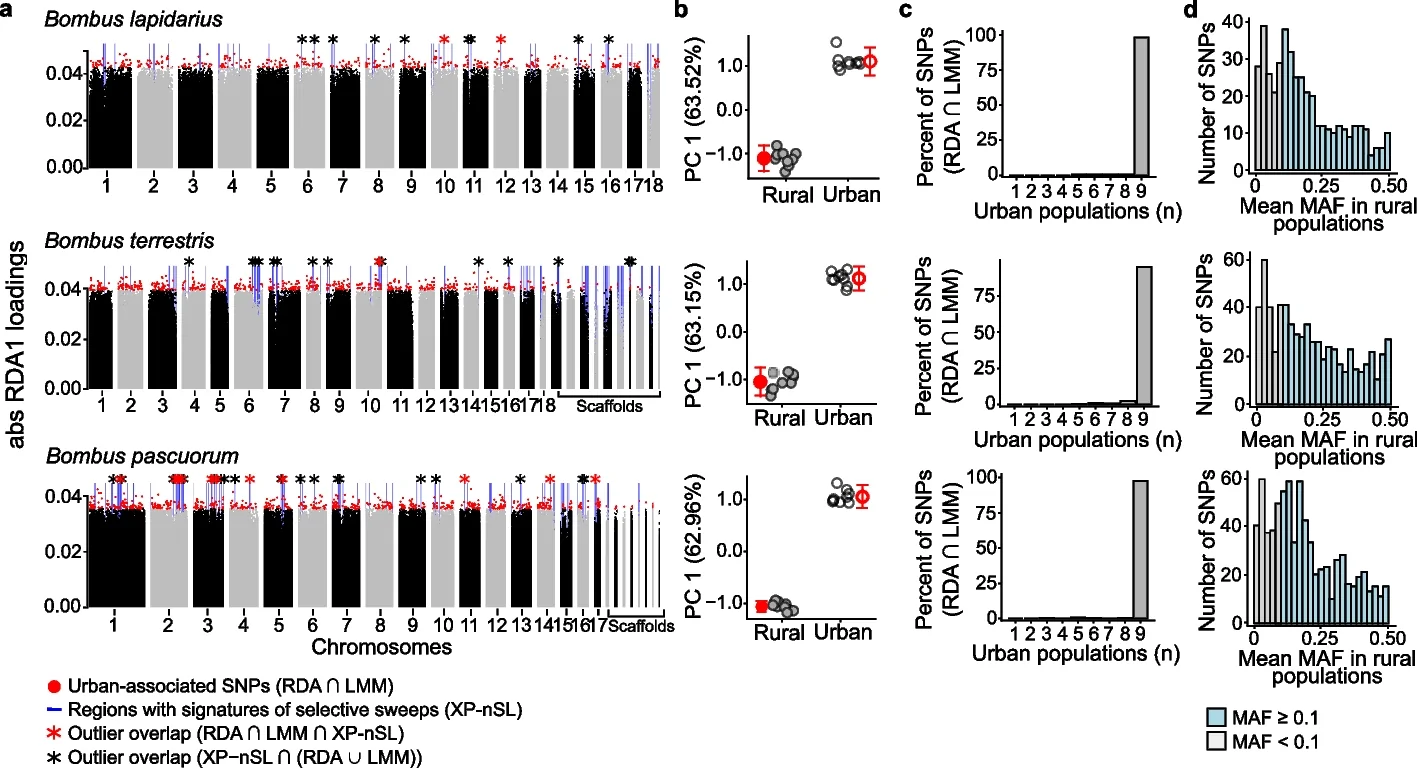
Genomic signals of divergence associated with urban bumble bee populations. **a** Manhattan plots showing absolute RDA1 scores across the genome. SNPs associated with urban environments identified jointly by redundancy analysis and the paired linear mixed model (RDA ∩ LMM) are highlighted in red. Blue horizontal bars indicate genomic regions exhibiting signatures of selective sweeps in urban populations detected using XP-nSL. Black asterisks denote regions where selective sweeps overlap with either GEA approach, whereas red asterisks denote overlap with SNPs jointly identified by RDA and LMM (RDA ∩ LMM). **b** Scores along the first principal component (PC1) from a PCA based on urban-associated SNPs (RDA ∩ LMM). Grey circles represent sampling sites; red points and error bars show model-estimated means ± SE. **c** Proportion of urban-associated SNPs (RDA ∩ LMM) for which the same allele was the minor allele across increasing numbers of the nine independent urban populations. **d** Histograms showing the distribution of mean minor allele frequencies (MAF) of urban-associated SNPs across rural populations. SNPs with mean rural MAF ≥ 0.1 are highlighted in cyan, indicating that both alleles were already segregating at appreciable frequencies in rural populations and are therefore consistent with standing genetic variation; SNPs with mean rural MAF < 0.1 are shown in grey

We next assessed concordance among methods. All SNPs identified by RDA were also detected by the paired LMM, yielding 451 shared urban-associated SNPs in *B. lapidarius*, 641 in *B. terrestris* and 763 in *B. pascuorum* (Fig. 3a). Of these shared SNPs (RDA ∩ LMM), 98.4% in *B. lapidarius*, 99.8% in *B. terrestris* and 99.0% in *B. pascuorum* were independently supported by LFMM analyses. In all three species, overlap between methods was significantly greater than expected by chance (hypergeometric tests, all *P* < 0.001; Additional file 2: Fig. S4), providing strong evidence that these loci are consistently associated with urban environments across multiple analytical approaches.

We further examined whether allele-frequency variation at urban-associated SNPs (RDA ∩ LMM) was associated with specific components of the urban environment. Because road density and land-surface temperature were strongly correlated (Pearson’s *r* = 0.89), they were evaluated in separate models, with PM_2_._5_ and NO_2_ retained as predictors in both. Associations with both land-surface temperature and road density were widespread across all three species. After FDR correction, land-surface temperature was significantly associated with 85.7% of urban-associated SNPs in *B. lapidarius*, 78.9% in *B. terrestris* and 77.6% in *B. pascuorum*. Road density was associated with 98.9%, 98.0% and 97.4% of urban-associated SNPs, respectively. In contrast, associations with PM_2_._5_ and NO_2_ were limited: no urban-associated SNPs were significantly associated with PM_2_._5_ after FDR correction, while only a small proportion were associated with NO_2_ (0% in *B. lapidarius*, 0.68% in *B. terrestris* and 0.31% in *B. pascuorum*). Thus, allele-frequency variation at urban-associated loci was predominantly associated with the correlated structural and thermal components of the urban environmental gradient, rather than with the measured air-pollution variables. Given the strong correlation between road density and land-surface temperature, however, their relative contributions cannot be disentangled, and these associations should not be interpreted as evidence for independent selective effects of either variable.

Urban-associated SNPs (RDA ∩ LMM) exhibited non-random spatial distributions within chromosomes. When chromosomes were divided into non-overlapping 100 kb windows, the number of outlier SNPs per window deviated significantly from random expectations in all species (*B. lapidarius*: G = 60.117, *P* < 0.001; *B. terrestris*: G = 218.667, *P* < 0.001; *B. pascuorum*: G = 207.214, *P* < 0.001), indicating heterogeneity in the spatial distribution of candidate loci within chromosomes. However, at the broader chromosome scale, candidate SNP counts were positively associated with total SNP number per chromosome (*B. lapidarius*: *t* = 3.820, *P* = 0.001; *B. terrestris*: *t* = 3.103, *P* = 0.007; *B. pascuorum*: *t* = 4.309, *P* < 0.001), indicating that much of the observed chromosome-level variation reflects underlying genomic opportunity. After accounting for total SNP number per chromosome, gene content showed no independent association with candidate SNP counts (*B. lapidarius*: *t* = − 0.654, *P* = 0.522; *B. terrestris*: *t* = 0.065, *P* = 0.949; *B. pascuorum*: *t* = 0.129, *P* = 0.899). Together, these results suggest that, although candidate loci exhibit some local spatial heterogeneity within chromosomes, urban-associated genomic divergence overall remains broadly distributed across the genome, consistent with a polygenic response to urbanisation.

Principal component analyses based on the shared urban-associated SNPs (RDA ∩ LMM) differentiated urban and rural populations in all three species despite minimal genome-wide population structure (Fig. 3b), consistent with repeated multivariate allele-frequency divergence across independent cities. Formal tests of parallelism further support this pattern. Across all three species, shared urban ecosystem effects were consistently stronger than site pair-specific interaction effects. Median ecosystem effect sizes were highly similar among species (*B. lapidarius*: η^2^ = 0.614; *B. terrestris*: η^2^ = 0.609; *B. pascuorum*: η^2^ = 0.615), whereas median ecosystem × site pair interaction effects were markedly lower (*B. lapidarius*: η^2^ = 0.184; *B. terrestris*: η^2^ = 0.187; *B. pascuorum*: η^2^ = 0.184). Moreover, allele-frequency shifts were highly concordant across replicated urban–rural contrasts, with 96%, 95% and 96.7% of candidate SNPs in *B. lapidarius*, *B. terrestris* and *B. pascuorum*, respectively, exhibiting the same allele as the minor allele across all nine urban populations (Fig. 3c). Together, these results provide strong evidence that urban-associated genomic divergence was highly parallel across independent urban systems rather than predominantly driven by city-specific genomic responses.

Overall, our results indicate consistent urban-associated genetic divergence in the face of high gene flow across multiple central European cities and multiple bumble bee species, as evidenced by parallel intraspecific allele-frequency shifts at the same loci. Given the near-panmictic population structure inferred in all three species, these repeated allele-frequency shifts are most plausibly explained by selection acting on standing genetic variation, rather than by the spread of adaptive alleles among urban populations via gene flow. Consistent with this interpretation, the majority of urban-associated SNPs were already segregating at appreciable frequencies in rural populations, with mean rural minor allele frequencies ≥ 0.1 at 67.6% of SNPs in *B. lapidarius*, 69.2% in *B. terrestris* and 70.5% in *B. pascuorum* (Fig. 3d). This highly consistent pattern across species is compatible with evolutionary responses arising largely from standing genetic variation, provided that contemporary rural populations approximate the ancestral genetic background. However, the precise selective dynamics underlying these patterns remain unresolved, including whether they reflect recent selection acting on large effective populations with limited migration, or strong recurrent selection operating on a largely panmictic gene pool. Nevertheless, as in other highly connected systems, the loci identified here likely represent genomic regions exhibiting elevated divergence associated with urban environments despite ongoing gene flow [27, 42, 44, 68, 75–79].

### Selective sweeps

**Fig. 4:**
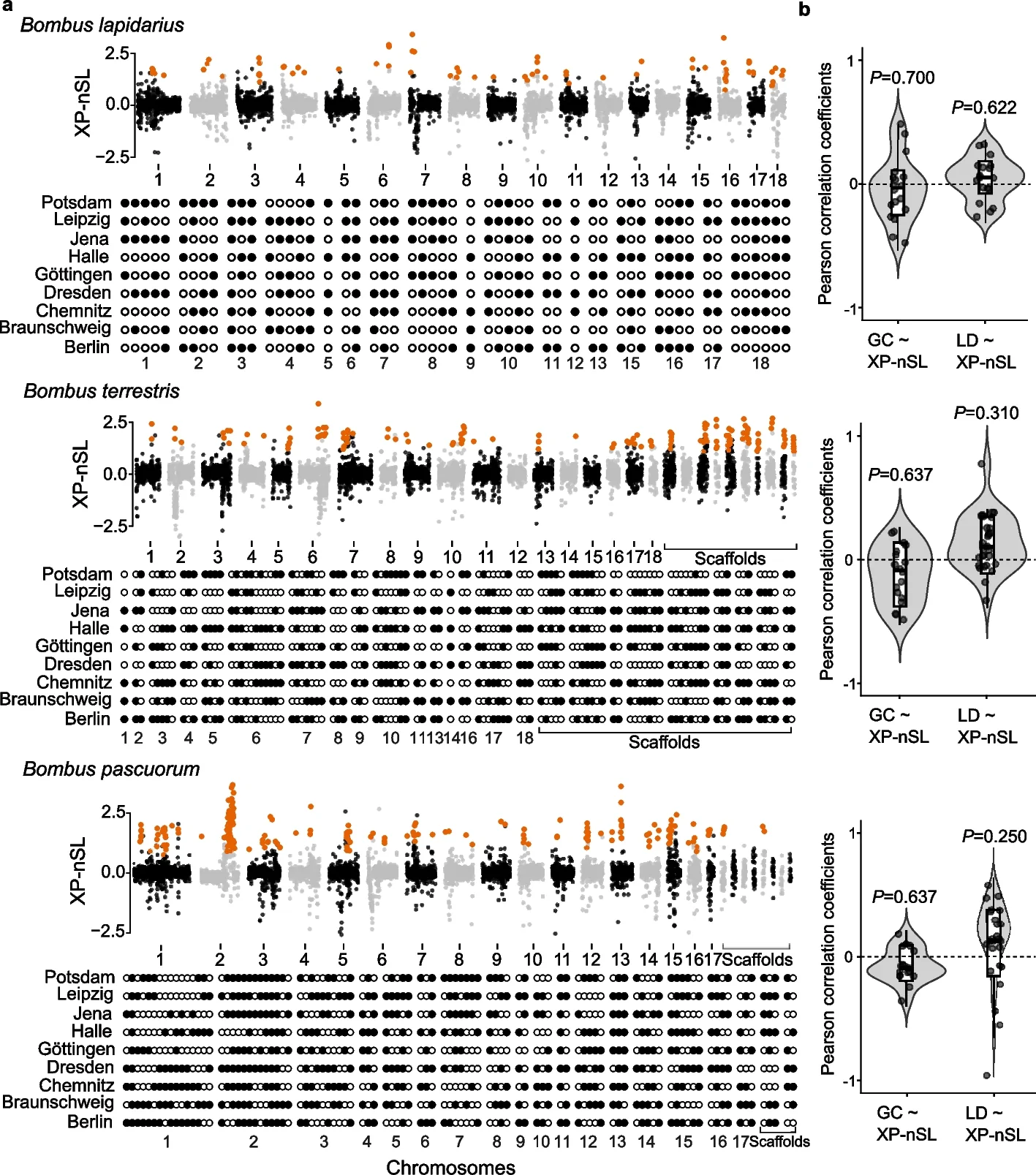
Selective sweep signals in urban bumble bee populations. **a** Manhattan plots showing genome-wide distributions of cross-population nSL (XP-nSL) scores calculated in non-overlapping 50 kb windows for urban–rural comparisons in *Bombus lapidarius*, *B. terrestris* and *B. pascuorum*. Positive XP-nSL values indicate stronger signatures of selection in urban populations. Orange points denote genomic windows identified as XP-nSL outliers (Z > 3) in four or more of the nine independent urban–rural population pairs, and therefore retained as robust, repeatedly detected selective sweep regions. Below each Manhattan plot, dot matrices indicate the presence (filled circles) or absence (open circles) of XP-nSL outlier windows in each individual city pair, illustrating the degree of replication across cities for each genomic region. **b** Violin plots showing the distribution of Pearson’s correlation coefficients (*r*) between XP-nSL scores and either linkage disequilibrium (LD) or intronic GC content across chromosomes. For each species, chromosome-specific correlation coefficients were transformed using Fisher’s *z*-transformation, and genome-wide significance was assessed by testing whether the mean *z* value differed from zero. Embedded boxplots indicate the median (vertical line), interquartile range (box) and data range (whiskers)

To identify signatures of positive selection associated with urban environments, we used the cross-population nSL statistic (XP-nSL). Genomic regions detected in four or more of the nine independent urban–rural comparisons were considered recurrent candidate sweep regions consistently associated with urban environments, based on a resampling-based null model indicating that such overlap was unlikely to arise by chance. Using this criterion, we identified 54 genomic regions in *B. lapidarius* (1.46% of the genome), 104 in *B. terrestris* (2.64% of the genome) and 99 in *B. pascuorum* (3.24% of the genome) exhibiting signatures of selective sweeps in urban populations (Figs. 3a and 4a; Additional file 1: Tables S11–S13). The large number of sweep regions detected across all three species, together with the extensive set of urban-associated SNPs distributed throughout the genome, is consistent with a polygenic genomic response to urban environments. To assess whether these sweep signals could be driven by non-adaptive processes such as linked selection in regions of low recombination, we tested for associations between XP-nSL scores and two proxies of recombination rate: linkage disequilibrium and intronic GC content, calculated in non-overlapping 50 kb windows. We detected no significant overall relationships between XP-nSL scores and either measure (Fig. 4b), suggesting that broad-scale variation in recombination rate and linked selection are unlikely to be major contributors to the observed genomic divergence between urban and rural populations. Selective sweep regions also showed repeated overlap with loci associated with urban environments identified by genome–environment association analyses (RDA or LMM; Fig. 3a). In *B. lapidarius*, 11 XP-nSL outlier regions overlapped with urban-associated SNPs, compared with 15 such regions in *B. terrestris* and 36 in *B. pascuorum* (Additional file 1: Tables S14–S16). In all three species, the observed degree of overlap significantly exceeded random expectations (one-sided binomial tests, all *P* < 0.001), supporting the inference that selection contributes to genomic divergence associated with urban environments.

Notably, in *B. pascuorum*, the strongest overlap between XP-nSL and urban-associated loci occurred within a contiguous six-megabase (6 Mb) region on chromosome 2 (Figs. 3a and 4a). Given the unusually high density of selective sweep signals in this region, we hypothesise that it may represent a polymorphic chromosomal inversion segregating among *B. pascuorum* populations. Chromosomal inversions can facilitate divergence in the face of gene flow by suppressing recombination, thereby increasing linkage among co-adapted alleles and reducing the effective migration of maladaptive haplotypes [79–83]. Such genomic architecture is therefore expected to generate strong, coincident signals between selective sweep scans and environment-associated loci. To explore this possibility, we used Asaph v.2.0 [84, 85], which detects large chromosomal inversions from SNP data using principal component analysis and PC–SNP association tests. This analysis revealed clear genetic clustering patterns consistent with the presence of a polymorphic inversion on chromosome 2 (Additional file 2: Fig. S5). Although confirmation using long-read or linked-read sequencing will be required to validate its structure and breakpoints, these results suggest that structural variation may contribute to urban-associated genomic divergence in *B. pascuorum*.

Although all three bumble bee species exhibited significant overlap between genomic regions showing signatures of selective sweeps (XP-nSL) and loci associated with urban environments, many urban-associated loci lacked classical sweep signatures, indicating that urban-driven genomic divergence is not restricted to strong, population-wide selective sweeps. Such partial concordance is expected, as sweep-based and genome–environment association (GEA) approaches capture complementary modes of selection [86]. XP-nSL is designed to detect population-wide positive selection, identifying genomic regions in which advantageous alleles have rapidly increased in frequency, often resulting in extended haplotype homozygosity and reduced genetic diversity in surrounding regions [87, 88]. In contrast, GEA approaches aim to identify loci exhibiting allele-frequency divergence associated with environmental variation, which may reflect divergent selection across environments rather than uniform fixation [87]. Consistent with these differences, overlap between XP-nSL and the paired LMM was consistently greater than overlap between XP-nSL and RDA. While this pattern is partly expected because all RDA candidates were nested within the larger paired LMM candidate set, it is also consistent with the different classes of genomic signals targeted by the two approaches. The paired LMM approach tests for repeated, directionally concordant allele-frequency differences between urban and rural populations across replicated site pairs, closely matching the population-wide selective scenarios to which sweep-based methods are most sensitive. In contrast, RDA is designed to detect coordinated, polygenic shifts across many loci, including subtle allele-frequency changes arising from soft sweeps or rapid responses from standing genetic variation. Such polygenic responses may not generate the strong haplotype-level signatures required for detection by selective sweep scans [89–91]. Urban environments are likely to impose heterogeneous and multifaceted selective pressures, favouring a combination of strong selection on specific genomic regions and more diffuse polygenic responses. Accordingly, only a subset of loci associated with urban environments are expected to coincide with classical sweep signatures. Similar patterns of incomplete yet informative overlap between sweep-based and environment-association approaches have been reported in other systems, underscoring the value of integrating complementary analytical frameworks to capture the full spectrum of genomic responses to environmental change [22, 26, 89, 92].

### Functional characterisation of candidate loci under selection

**Fig. 5:**
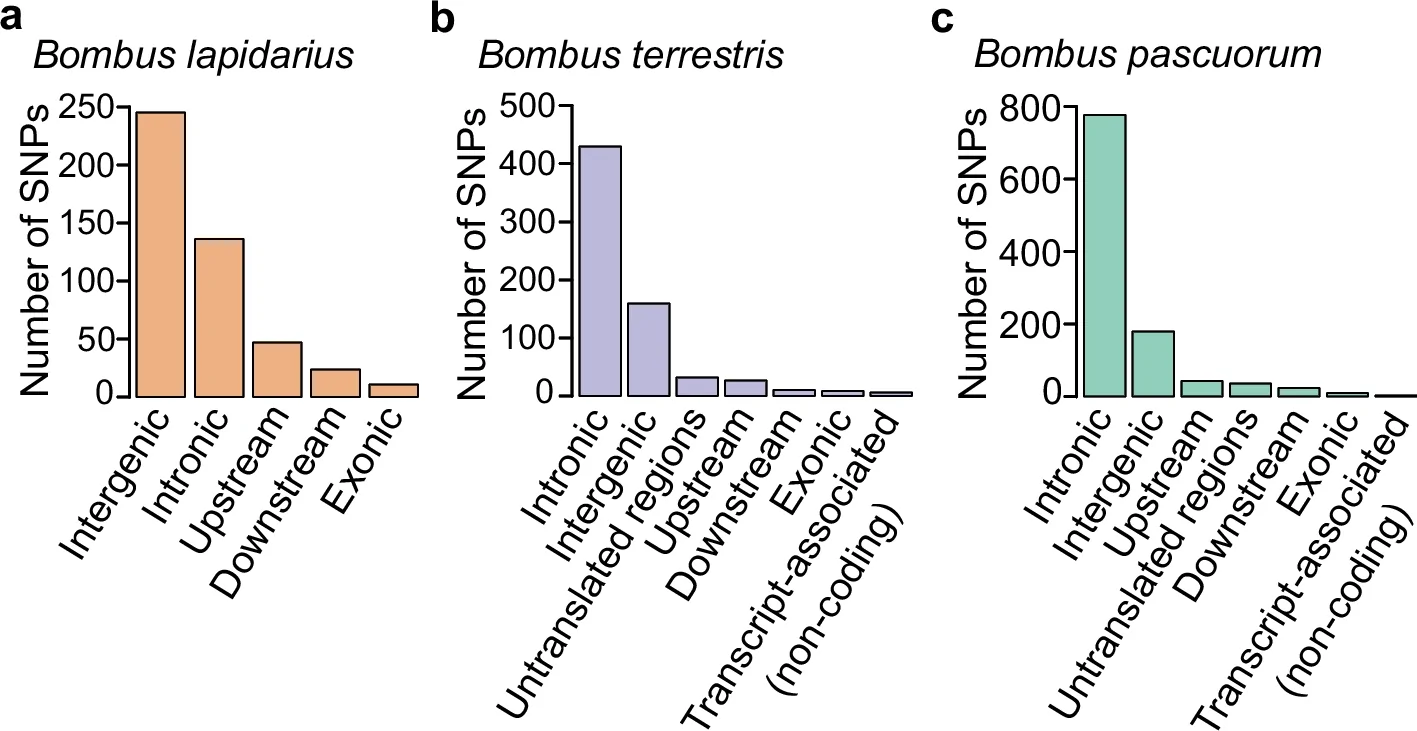
Genomic annotation of urban-associated SNPs. Genomic annotation of urban-associated SNPs comprising RDA ∩ LMM loci together with additional GEA candidate SNPs overlapping XP-nSL selective sweep regions, based on SnpEff predictions for **a** *Bombus lapidarius*, **b** *B. terrestris* and **c** *B. pascuorum*

Urban-associated SNPs—defined as SNPs jointly identified by RDA and the paired LMM (RDA ∩ LMM), together with additional LMM candidate SNPs overlapping with XP-nSL selective sweep regions—showed broadly similar genomic distributions across species. In *B. lapidarius*, most urban-associated SNPs were located in intergenic regions (52.9%), followed by intronic variants (29.4%) and upstream gene regions (10.2%) (Fig. 5a; Additional file 1: Table S17). Urban-associated SNPs in *B. terrestris* and *B. pascuorum* were predominantly intronic (63.9% and 72.2%, respectively), with smaller proportions occurring in intergenic regions (23.6% and 16.7%, respectively) and upstream regions (4.0% and 4.1%, respectively) (Fig. 5b and c; Additional file 1: Tables S18 and S19). Across all three species, only small fractions of SNPs were located in protein-coding exons (1.3–2.4%), indicating that genomic divergence associated with urban environments is largely concentrated in non-coding regions. Variants located in intergenic, upstream and intronic regions may influence regulatory elements such as promoters, enhancers and non-coding RNAs, as well as affect splicing and other regulatory processes within genes. Through such mechanisms, regulatory variation could contribute to consistent genomic divergence associated with urban environments without requiring widespread changes to protein-coding sequences. A predominance of non-coding over coding variants among loci associated with environmental divergence has been reported in numerous whole-genome studies of insects [76, 93–95] as well as in other taxa including birds [35], fish [96, 97], sheep and goats [98] and plants [99]. Together, these patterns are consistent with regulatory, polygenic genomic responses playing a central role in divergence associated with complex and heterogeneous environments such as cities.

**Fig. 6:**
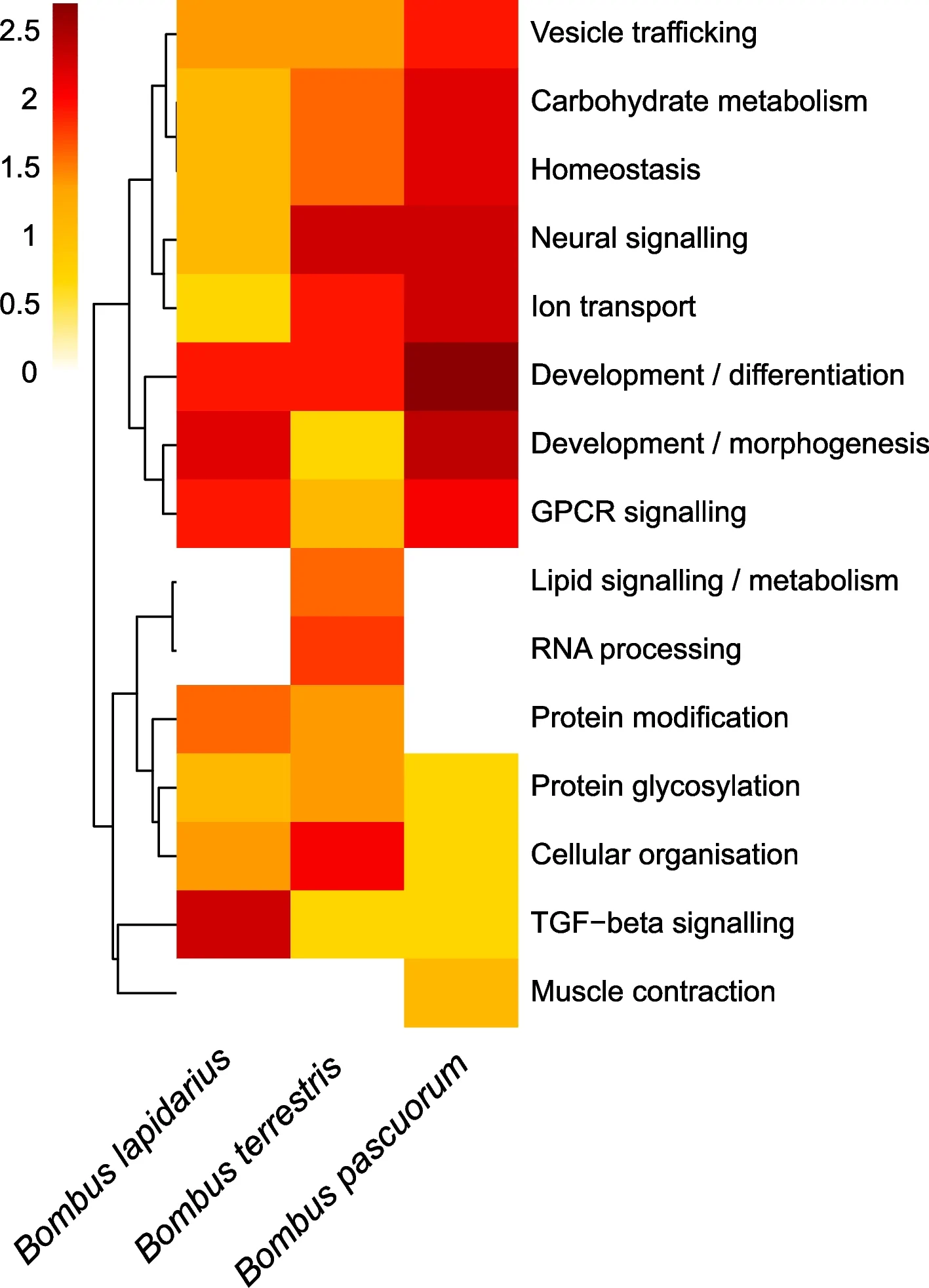
Functional convergence of urban-associated candidate genes across three bumble bee species. Heatmap showing semantic clusters of enriched Biological Process Gene Ontology terms associated with urban-associated candidate genes in *Bombus lapidarius*, *B. terrestris* and *B. pascuorum*. GO terms were clustered using semantic similarity and then manually grouped into simplified biological themes. Cell colours represent log_10_-transformed counts of GO terms assigned to each biological cluster in each species, with warmer colours indicating a larger number of enriched GO terms. The row dendrogram shows hierarchical clustering of biological clusters based on their distribution across species

Using genome annotations, we identified numerous candidate genes occurring within ± 10 kb of urban-associated SNPs across all three bumble bee species (Additional file 1: Tables S20–S22). This yielded 331 candidate genes in *B. lapidarius*, 438 in *B. terrestris* and 522 in *B. pascuorum*. GO enrichment analyses using the topology-aware (*elim*) algorithm identified significantly enriched terms across all three GO ontologies in each species (Fig. 6; Additional file 1: Tables S23-25). Complementary FDR-adjusted enrichment analyses showed significant overlap with the GO terms identified by the primary *elim* analysis (one-sided hypergeometric tests, BH-adjusted *P* < 0.001 for all species). Sensitivity analyses further indicated that GO enrichment patterns were generally robust to SNP-to-gene assignment window size, with significant overlap among enriched GO-term sets across 0 kb, ± 5 kb and ± 10 kb assignments under both the *elim* and FDR-corrected analyses (one-sided hypergeometric tests, BH-adjusted *P* < 0.001 for all comparisons; Additional file 1: Tables S23–S25). At the broad functional level, themes related to neural function, development, signalling and stress response were consistently recovered across window sizes.

In *B. lapidarius*, urban-associated candidate genes were enriched for functions related to lipid and fatty-acid metabolism, neural and synaptic processes, cellular signalling and developmental regulation (Fig. 6; Additional file 1: Tables S20 and S23). Enriched pathways included fatty-acid β-oxidation, lipid homeostasis and glycosyl metabolism, alongside candidate genes such as aquaporin and sestrin-1, previously implicated in responses to thermal, pollution and pesticide stress in bees [100–102]. Neural and synaptic functions, including synapse organisation, synaptic vesicle cycling and neurotransmitter transport, were also prominent. Notably, genes such as neurexin-1 and lachesin have previously been associated with responses to environmental stressors in bees and other insects [68, 100, 103–105], suggesting that neurophysiological pathways may represent recurrent targets of anthropogenic environmental pressures. Additional enrichment of Notch, BMP and TGF-β signalling, together with cytoskeletal organisation and morphogenesis, further implicated developmental and regulatory processes. Overall, these findings suggest that urban-associated genomic divergence in *B. lapidarius* involves metabolic, neural and regulatory pathways, several of which have previously been linked to environmental stress responses.

In *B. terrestris*, urban-associated candidate genes were enriched for functions related to neural and sensory processing, neuromuscular development, metabolic regulation, immunity and developmental processes (Fig. 6; Additional file 1: Tables S21 and S24). Neural-associated pathways included axon guidance, synaptic transmission, receptor localisation, ion transport and calcium signalling, with candidate genes such as neurobeachin, glutamate receptors and acetylcholinesterase previously implicated in responses to temperature variation, pesticide exposure and altered physiological conditions [68, 104, 106, 107]. Other candidates involved in neural development, including Drl and Ror, have been linked to temperature-associated genomic variation in bumble bees [42, 68], suggesting potential overlap between climatic and urban selective pressures. Enrichment of sensory pathways involving odorant, dopamine and octopamine receptors further points to possible divergence in environmental perception relevant to foraging and navigation. Neuromuscular and cytoskeletal functions were also prominent, including titin, previously implicated in genomic responses to air pollution, pesticide exposure and other environmental stressors in bees [95, 102, 107]. Additional enrichment of lipid and glucose metabolism, cytochrome P450-associated processes, redox homeostasis, immune function and autophagy suggests that urban-associated divergence encompasses pathways potentially involved in detoxification, physiological stress responses and host defence [68, 100, 101, 107]. Developmental regulators previously associated with climatic gradients in bumble bees were also represented [67]. Together, these findings indicate that genomic divergence in *B. terrestris* involves multiple neural, sensory, metabolic, immune and regulatory pathways previously implicated in responses to environmental stressors relevant to urban habitats.

In *B. pascuorum*, urban-associated candidate genes were enriched for neural and synaptic function, ion and calcium transport, neuromuscular processes, oxidative stress responses, sensory perception and immune regulation (Fig. 6; Additional file 1: Tables S22 and S25). Oxidative stress and redox homeostasis pathways included candidate genes such as peroxidasin-like and oxidation resistance protein 1, previously associated with environmental and physiological stress responses in bees [68, 108]. Metabolic and nutrient-processing functions were also represented, consistent with previous evidence of energetic responses to thermal and pesticide stress [101, 104]. Neural and synaptic candidates included neurexin-1, neurobeachin, neuroligin-4 and Drl, with similar loci previously implicated in responses to temperature gradients, pesticide exposure and air pollution [68, 102, 109], suggesting potential convergence in neurophysiological responses to different environmental stressors. Sensory pathways involving olfactory and gustatory perception were also enriched, consistent with earlier studies reporting receptor-associated responses to pesticide and pollution exposure [102, 104]. Neuromuscular functions included muscle contraction, calcium release and excitation–contraction coupling, with candidates such as titin and the ryanodine receptor. Notably, titin has repeatedly been implicated in responses to diesel exhaust particles and pesticide exposure in bees [102, 107]. Finally, enrichment of immune- and host-defence pathways, including hymenoptaecin-like and defensin-related genes, parallels previous findings linking immune and microbiome-associated processes to pesticide exposure and physiological stress [100, 104, 107, 109, 110]. Together, these results suggest that urban-associated genomic divergence in *B. pascuorum* involves stress-response, neural, sensory, neuromuscular and immune pathways, several of which have previously been implicated in responses to anthropogenic environmental pressures.

Despite differences in the specific candidate genes and enriched functions among species, several broad functional themes recurred, including physiological stress responses, neural and behavioural processes, metabolism, developmental regulation and immunity (Fig. 6; Additional file 2: Fig. S6; Additional file 1: Tables S23–S25). These patterns are consistent with our environmental association analyses, which showed particularly strong covariation between allele frequencies at urban-associated loci and land-surface temperature and road density. Moreover, the implicated pathways correspond to traits and physiological processes previously shown to differ across urban gradients or between urban and rural bee populations, including oxidative stress [55], thermal tolerance [111], parasitism and immunometabolism [52, 58, 112–114], body size [16, 115], and brain size, learning and memory [116, 117]. Similar functional pathways have also been implicated in population genomic and gene expression studies of bees and other insects exposed to environmental stressors [42, 68, 100–110, 118]. More broadly, our findings are consistent with studies in other animal taxa reporting urban-associated genomic variation involving cognition, immunity, stress physiology and behaviour [20, 24, 26, 119]. Together, these results suggest that although the specific genomic targets of urban-associated divergence are largely species-dependent, urban environments may impose sufficiently recurrent selective pressures to generate convergence at the level of broader biological functions.

### Parallels across species

We detected substantial overlap in urban-associated candidate genes among bumble bee species, indicating partial convergence in genomic responses to urban environments. Pairwise comparisons revealed that *B. lapidarius* and *B. terrestris* shared 25 orthologous candidate genes, *B. lapidarius* and *B. pascuorum* shared 29 orthologous genes, and *B. terrestris* and *B. pascuorum* shared 120 orthologous genes (Additional file 2: Fig. S6; Additional file 1: Tables S26–S28). In all pairwise comparisons, the observed overlap significantly exceeded random expectations based on the shared gene universe (one-sided hypergeometric tests, *P* < 0.01; Additional file 2: Fig. S6). These patterns were broadly robust to SNP-to-gene assignment window size, with many of the same orthologous genes recovered across 0 kb, ± 5 kb and ± 10 kb windows (Additional file 1: Tables S26–S28).

Across all three species, six candidate genes were jointly identified as urban-associated (Additional file 2: Fig. S6; Additional file 1: Table S29). Permutation tests based on the shared gene universe across all three species confirmed that this three-way overlap was significantly greater than expected by chance (one-sided permutation test, *P* < 0.001; Additional file 2: Fig. S6), indicating non-random convergence across species at a small subset of loci. Although most candidate genes were species specific, these six shared loci represent plausible genomic targets repeatedly associated with urban environments. Among these genes, several have well-established biological functions potentially relevant to urban-associated selective pressures (Additional file 1: Tables S29 and S30), including *tensin-1*, leucine-rich repeat-containing protein 4C-like (LRRC4C-like) and venom dipeptidyl peptidase 4 (*DPP4*). *Tensin-1* is a focal-adhesion scaffolding protein that links integrins to the actin cytoskeleton, making it a strong candidate for urban-associated shifts in cell adhesion, tissue mechanics and locomotor or flight performance—traits likely challenged by habitat fragmentation, altered airflow and navigation around built structures. LRRC4C-like belongs to a protein family implicated in neuronal development, cell adhesion and neural connectivity and has previously been identified among candidate genes associated with temperature seasonality in bumble bees [68]. This suggests that neural development and cell-adhesion-related processes may represent recurrent components of genomic responses to urban and climatic environmental variation. Finally, *DPP4* encodes a protease involved in peptide processing and immune or physiological regulation, making it a biologically plausible candidate under urban-associated shifts in pathogen exposure, microbiome composition and chronic stress. Consistent with this interpretation, *DPP4* has been identified as a component of *B. pascuorum* venom and shown to vary along environmental gradients of temperature and humidity, indicating sensitivity to abiotic conditions influencing defensive physiology [120].

Despite limited overlap at the level of individual genes, Gene Ontology (GO) enrichment analyses provided clear evidence for functional convergence in urban-associated genomic responses across species (Fig. 6). Semantic similarity analyses of enriched GO terms, calculated using the Wang method and summarised using the best-match average (BMA), were conducted within GO ontologies (biological process, molecular function and cellular component). These analyses revealed moderate to high functional similarity between species (mean Wang similarity ≈ 0.51), with observed similarity scores significantly greater than expected under permutation-based null models (*P* < 0.001). Together, these results indicate that urban-associated genomic divergence consistently targets similar functional pathways across species, even when the specific underlying genes differ.

## Conclusions

Our study shows that replicated environmental contrasts can generate predictable genomic divergence across independent populations and species, even in the presence of high gene flow. Across three bumble bee species and nine urban–rural population pairs, we detected consistent allele-frequency shifts at shared loci, revealing parallel genomic responses to urban environments despite negligible genome-wide population structure. These patterns indicate that strong and recurrent environmental pressures can overcome the homogenising effects of connectivity and produce repeated genomic signatures of divergence, with candidate-locus allele frequencies strongly covarying with correlated structural and thermal components of the urban environmental gradient. Urban-associated genomic variation was highly polygenic, broadly distributed throughout the genome, and predominantly concentrated in non-coding regions, highlighting regulatory variation as a central component of genomic responses to complex environmental change. Although most candidate genes were species specific, convergence occurred at the level of biological function, with repeated enrichment of pathways related to stress physiology, behaviour, metabolism and immunity. Together, these findings indicate that rapid anthropogenic environmental change can shape genomic architecture in predictable ways, involving coordinated allele-frequency shifts in non-coding regions with potential regulatory roles and convergence in similar functional pathways rather than repeated changes at identical coding loci. By integrating replicated urban–rural contrasts with whole-genome data and complementary analytical approaches, this study establishes a comparative framework for identifying consistent genomic divergence associated with anthropogenic environments. Future work linking urban-associated genomic variation to gene expression, phenotype and fitness will be important for clarifying the biological mechanisms underlying these patterns. Extending similar comparative genomic analyses across additional taxa and environmental contexts will help determine the generality of parallel divergence under rapid environmental change.

## Methods

### Study species

We focused on three co-occurring bumble bee species: the red-tailed bumble bee *B. lapidarius* (Linnaeus, 1758), the buff-tailed bumble bee *B. terrestris* (Linnaeus, 1758) and the common carder bee *B. pascuorum* (Scopoli, 1763). All three species are widespread and abundant across much of Europe, including the study region, and are important pollinators of both crops and wild flowering plants [52, 54, 61, 121]. Although *B. terrestris* is commercially traded, there is little evidence that the use of managed colonies has affected the population genomic structure of wild conspecifics [122, 123].

The three species differ in key ecological traits, including body size, nesting biology and dietary specialisation. Queens and workers of *B. lapidarius* and *B. terrestris* are similar in size and larger than those of *B. pascuorum* [124]. Both *B. lapidarius* and *B. terrestris* typically nest in subterranean cavities, whereas *B. pascuorum* nests close to or on the soil surface in dense vegetation, grass tussocks or leaf litter [61]. In addition, *B. pascuorum* has a narrower trophic niche and preferentially exploits flowers with deep corolla tubes, while *B. lapidarius* and *B. terrestris* are highly polylectic and show substantial dietary overlap [63]. These ecological differences among closely related species provide a comparative framework for testing whether genomic responses to urban environments are consistent across species or instead shaped by species-specific traits and habitat requirements.

### Study design and sample collection

Female workers of *B. lapidarius*, *B. terrestris* and *B. pascuorum* were collected between June and August 2014 from 18 sampling sites comprising nine German cities (Berlin, Braunschweig, Chemnitz, Dresden, Göttingen, Halle, Jena, Leipzig and Potsdam) and nine paired rural locations (Fig. 1). Urban sampling sites consisted of public green spaces (parks and gardens) located near city centres and surrounded by dense built infrastructure (Fig. 1; Additional file 1: Table S31). Paired rural sites were selected using land-cover data from Geofabrik GmbH (http://www.geofabrik.de) and Quantum GIS v3.16.3 to be dominated, within a 1 km radius, by semi-natural, forest and agricultural land typical of rural landscapes in Central Europe (Fig. 1; Additional file 1: Table S32).

To ensure independence among sampling locations, urban sites were separated by a minimum distance of 20 km. Each rural site was located 10–40 km (mean = 25 km) from its paired urban site and more than 25 km from all other sites (Fig. 1). These distances exceed the typical foraging and dispersal ranges of the study species [69, 70, 73]. All sites were selected to represent flower-rich habitats within their respective landscape types, allowing us to focus on contrasts at the landscape scale rather than variation driven by local floral resource availability.

Although sampling along continuous urbanisation gradients is commonly used to investigate urban effects, such designs often concentrate sampling effort at intermediate levels of urbanisation, potentially reducing statistical power to detect genetic–environment associations (unless responses to intermediate conditions are of specific interest) [125]. In contrast, paired urban–rural designs reduce confounding effects arising from isolation by distance and spatially structured gene flow, thereby increasing power to detect differences in genetic diversity and signatures of divergent selection associated with urban environments [126, 127]. Accordingly, our sampling strategy was designed to maximise the number of independent urban–rural comparisons across the study region.

All analyses were based on a categorical classification of sampling sites as urban or rural. To verify that this classification captured consistent environmental contrasts, we quantified independent measures of urban intensity and pollution at each site. Specifically, we estimated the proportion of impervious surface cover (buildings and roads) within a 1 km radius, derived from land-cover data provided by Geofabrik GmbH, land-surface temperature (°C; MODIS data, 2009–2014, spring and summer daytime, 1 km radius), and atmospheric concentrations of fine particulate matter (PM_2_._5_; µg m⁻^3^) and nitrogen dioxide (NO_2_; µg m⁻^3^) using the Copernicus European air-quality dataset (2013–2014, spring and summer, 10 km resolution). Differences between urban and rural sites were tested using linear mixed-effects models (LMMs) with site pair included as a random effect, implemented in the *nlme* package v.3.1–164 [128].

Using hand nets, we collected a total of 624 worker bees across the three species (Additional file 1: Table S33), comprising 200 B*. lapidarius*, 202 B*. terrestris* and 222 B*. pascuorum* female individuals. All individuals were sampled within 250 m of the centre of each sampling site. Specimens were preserved in 100% ethanol and stored at -20 °C until DNA extraction. All field sampling was conducted in 2014 in accordance with local regulations and relevant ethical guidelines.

### DNA extraction, whole genome sequencing and SNP genotyping

High-molecular-weight genomic DNA was extracted from thoracic muscle tissue using proteinase K digestion in CTAB buffer, followed by chloroform:isoamyl alcohol extraction (24:1). DNA concentration and quality were assessed using a Qubit 3.0 fluorometer (Thermo Fisher Scientific, Waltham, MA, USA) and an Epoch spectrophotometer (BioTek, Winooski, VT, USA). For each individual, 500 ng of DNA was standardised to a concentration of 15 ng µl⁻^1^. DNA libraries were prepared using the Illumina DNA Prep kit and sequenced by SNPsaurus (Eugene, OR, USA) on an Illumina NovaSeq 6000 S4 lane, generating 150-bp paired-end reads and approximately 5.5 Gbp of sequence data per individual. Read numbers were high and broadly consistent across species and populations (*B. lapidarius*: mean = 16,888,181; SD = 4,057,381; *B. terrestris*: mean = 18,299,295; SD = 5,382,363; *B. pascuorum*: mean = 19,146,370; SD = 5,190,723; Additional file 1: Tables S34–S36).

Raw sequence data were initially assessed for quality using FastQC v 0.12.0 [129]. Adapter removal, quality trimming and filtering were performed using BBDuk (BBMap v38.91; parameters: ktrim = r, k = 17, mink = 8, hdist = 1, tpe, tbo, qtrim = rl, trimq = 10). Cleaned reads were mapped to species-specific reference genomes (*B. lapidarius*: iyBomLapi1.1 [130, 131]; GCA_964186655.1; *B. terrestris*: iyBomTerr1.2 [132, 133]; GCF_910591885.1; *B. pascuorum*: iyBomPasc1.1 [134, 135]; GCA_905332965.1) using bwa-mem2 v 2.2.1 with default parameters [136]. PCR duplicates were marked using GATK v 4.2.2.0 [137].

Sequencing depth and coverage across the genome were analysed using SAMtools v. 1.7 [138]. Mean sequencing depth and coverage were high and consistent across species and populations (sequencing depth: *B. lapidarius*: mean = 11.01×, SD = 2.54; *B. terrestris*: mean = 10.90×, SD = 3.16; *B. pascuorum*: mean = 12.14×, SD = 3.24; genome coverage: *B. lapidarius*: mean = 97.70%, SD = 0.70%; *B. terrestris*: mean = 97.11%, SD = 0.98%; *B. pascuorum*: mean = 98.54%, SD = 0.65%; Additional file 1: Tables S34–S36), indicating robust coverage suitable for reliable variant detection. Variant calling was performed using BCFtools v. 1.23 [139]. Genotype likelihoods were generated from duplicate-marked BAM files using bcftools mpileup, excluding reads with mapping quality < 20 (-q 20) and bases with base quality < 30 (-Q 30). Variant and invariant sites were called using bcftools under a multiallelic calling model (-m), without restricting output to variant sites (i.e. without -v). Resulting VCF files were merged across chromosomes and processed using VCFtools v.0.1.16 [140] for downstream filtering.

To remove low-quality and sparsely genotyped loci, an initial filtering step retained SNPs genotyped in at least 50% of individuals (--max-missing 0.5), with a minimum site quality score of 30 (--minQ 30) and a minor allele count ≥ 3 (--mac 3). To limit the influence of low-coverage genotypes, a minimum per-individual genotype depth of three reads was enforced (--minDP 3). Genotyping error rates associated with low sequencing depth (≤ 5×) were assessed using the ErrorCount.sh script from the dDocent pipeline [141], which estimates expected error rates based on binomial probabilities. Estimated error rates were negligible, ranging from 0.005–0.02% in *B. lapidarius*, 0.006–0.03% in *B. terrestris*, and 0.004–0.02% in *B. pascuorum*, indicating high genotyping accuracy.

Individuals with > 50% missing data were identified using the --missing-indv function in VCFtools and subsequently removed from the dataset. For analyses of genetic diversity, population divergence and population structure, additional filtering retained high-confidence SNPs genotyped in all individuals (--max-missing 1), with a minor allele frequency ≥ 0.05 (--maf 0.05), mean site depth between 5 and 60 reads (--min-meanDP 5; --max-meanDP 60), and exclusion of indels (--remove-indels). For selection analyses, filtering thresholds were relaxed to retain SNPs genotyped in at least 75% of individuals (--max-missing 0.75), while applying the same allele frequency, depth and variant-type filters. After initial filtering, realised genotype missingness across retained datasets remained low (*B. lapidarius*: 2.91%; *B. terrestris*: 3.83%; *B. pascuorum*: 2.01%). Individual-level missingness was also generally low (mean = 2.90% in *B. lapidarius*; mean = 3.83% in *B. terrestris*; mean = 2.00% in *B. pascuorum*; Additional file 1: Tables S34–S36). Retained SNPs showed consistently high realised coverage after filtering, with SNP mean-depth distributions approximately 10× across all three species (Additional file 2: Fig. S7).

To minimise confounding effects of family structure, pairwise relatedness among individuals was estimated using VCFtools --relatedness2 [142]. Pairs of individuals with relatedness values > 0.177, indicative of second-degree or closer relatives, such as workers originating from the same colony [67, 142], were identified, and only one individual per related pair was retained. In such cases, the individual with the highest mean sequencing depth was preferentially kept for downstream analyses. This procedure resulted in the removal of 20, 16 and 27 individuals from the *B. lapidarius*, *B. terrestris* and *B. pascuorum* datasets, respectively. Post-filtering relatedness analyses confirmed that kinship was low across populations, with maximum pairwise relatedness values remaining substantially below the threshold used to identify close relatives (0.07 in *B. lapidarius*, ≤ 0.01 in *B. terrestris* and 0.08 in *B. pascuorum*) and showing no systematic differences between urban and rural populations (Additional file 1: Tables S37–S39).

For analyses of population divergence and structure, SNPs were additionally filtered for departures from Hardy–Weinberg equilibrium within populations using VCFtools --hardy, removing loci with a *P* < 0.01 in any population. For analyses of genetic diversity, population divergence and population structure, linkage disequilibrium was reduced by pruning SNPs using PLINK v1.9 [143] with the parameters --indep-pairwise 50 5 0.5, removing SNP pairs with pairwise linkage disequilibrium (r^2^) > 0.5 within a 50-SNP sliding window. This threshold was selected to reduce strong local linkage while retaining broad genome-wide representation across the genome. To evaluate robustness, we additionally repeated the population structure analyses using a stricter pruning threshold (--indep-pairwise 50 5 0.2), which produced qualitatively similar population structure patterns (Additional file 2: Fig. S8).

Different filtering thresholds were applied depending on the analytical objective, reflecting the distinct statistical assumptions and sensitivities of diversity, population structure and selection analyses and following current best-practice recommendations in population genomics [144]. After all filtering steps and relatedness checks, the final datasets comprised 172 individuals (mean per population = 10, SD = 2) and 157,868 SNPs for genetic diversity analyses, 148,057 SNPs for population structure analyses, and 1,714,239 SNPs for selection analyses in *B. lapidarius*; 171 individuals (mean = 10, SD = 1) and 75,586 SNPs (genetic diversity), 63,980 SNPs (population structure), and 2,251,873 SNPs (selection) in *B. terrestris*; and 181 individuals (mean = 10, SD = 1) and 528,194 SNPs (genetic diversity), 497,114 SNPs (population structure), and 3,215,849 SNPs (selection) in *B. pascuorum* (Additional file 1: Table S33).

### Genetic diversity, genetic divergence and population genetic structure

Genome-wide genetic diversity was quantified as expected heterozygosity (*H*_exp_) for each sampling site using the R package *dartR* v. 2.9.7 [145]. To obtain estimates that were both genome-wide [146] and unbiased with respect to sample size, we calculated unbiased adjusted expected heterozygosity (*adjH*_exp_). This required quantifying the total number of callable loci per population from an all-sites VCF generated using bcftools call without the -v option, thereby retaining both polymorphic and invariant sites. Adjusted heterozygosity was calculated as:

1 $$adj{H}_{EXP}={H}_{EXP}\times \frac{2n}{2n-1}\times \frac{{\mathrm{n}}_{\mathrm{polymorphic}\;\mathrm{loci}}}{{\mathrm{n}}_{\mathrm{all} \;\mathrm{loci}}}$$

where *n* is the number of sampled individuals per population. The first correction term $\frac{2n}{2n-1}$ ​​ accounts for bias due to finite sample size, yielding an unbiased estimator of expected heterozygosity (*uH*_EXP_) [145]. The second correction term adjusts for the exclusion of invariant loci, preventing overestimation of heterozygosity when only polymorphic markers are considered and ensuring that diversity estimates reflect genome-wide variation [145, 146]. Contemporary effective population size trajectories were estimated to compare recent demographic patterns between urban and rural populations using the linkage disequilibrium-based method implemented in the software GONE [147]. Because sample sizes within individual local populations were limited for robust demographic inference, individuals were grouped by ecosystem type (urban versus rural) within each species prior to analysis. GONE was run using default parameters and assuming a recombination rate of 8.7 cM/Mb based on *Bombus terrestris* [148]. Because individuals originated from multiple geographically distinct urban–rural population pairs, inferred trajectories should be interpreted cautiously and primarily as qualitative comparisons of recent demographic patterns between ecosystem types rather than precise estimates of absolute effective population size. Results are presented for generations 10–200, given the greater uncertainty associated with estimates from the most recent and most distant generations in linkage disequilibrium-based demographic reconstructions [149, 150].

To assess the sensitivity of *uH*_EXP_ estimates to sampling effort, we conducted a subsampling analysis using *B. lapidarius*, the species with the smallest overall population sample sizes in our dataset. Five populations with the largest available sample sizes were selected to maximise the range of subsampling levels and individuals were randomly subsampled without replacement across a range of sample sizes. For each sample size, 100 independent subsampling replicates were performed and *uH*_EXP_ was recalculated for each subset using *dartR* [145]. To formally determine the minimum sample size required for stable estimates, we implemented a convergence-based analysis. For each population, the distribution of *uH*_EXP_ at the largest available sample size was taken as the reference (“plateau”). At each smaller sample size, we calculated the median heterozygosity across subsampling replicates and compared it with the plateau median. A bootstrap procedure with 2,000 iterations was used to generate 95% confidence intervals (CIs) for the difference in medians by resampling both the target and plateau distributions. Convergence was defined as the smallest sample size at which the 95% CI of the difference lay entirely within pre-specified equivalence bounds (Δ = 0.0002, 0.0003, 0.0005 and 0.001). This framework allowed us to identify the sample size at which heterozygosity estimates became statistically indistinguishable from plateau values under increasingly stringent convergence criteria and to assess whether smaller subsets yield representative estimates of population-level genetic diversity after correction for sample size. Across all populations, *uH*_EXP_ stabilised rapidly (Additional file 2: Fig. S9), with convergence reached at sample sizes of 2–5 individuals under a moderate equivalence threshold (Δ = 0.0005) (Additional file 2: Fig. S9). These results indicate that *uH*_EXP_ estimates were relatively stable at the sample sizes represented in our dataset.

We tested whether genetic diversity differed between urban and rural populations using LMMs implemented in the *nlme* R package [128]. Ecosystem type (urban versus rural) was included as a fixed effect, and site pair was included as a random intercept to account for non-independence between paired populations. To account for variation in local habitat quality, we included local flowering plant richness—quantified using ten randomly placed 1 m^2^ quadrats per site—and the proportion of green cover (grassland, meadows, scrub vegetation, botanical gardens, public parks, and allotments) within a 1 km radius as covariates (Additional file 1: Tables S31 and S32). The response variable was *adjH*_EXP_.

Multicollinearity among predictors was assessed using variance inflation factors (VIFs), with a threshold of three used to identify potential issues [151]; all predictors met this criterion (VIF < 3). Spatial autocorrelation in model residuals was tested using Moran’s I implemented in the *ape* R package [152] and no significant spatial structure was detected (*P* > 0.05). To assess the robustness of the observed urban–rural differences in genetic diversity, we conducted a permutation test based on the fitted mixed-effects model. Estimated marginal means and pairwise contrasts for ecosystem type were obtained using the *emmeans* R package [153]. Ecosystem labels were randomly permuted 1,000 times, with the mixed-effects model refitted to each permuted dataset. For each permutation, the *t*-statistic associated with the ecosystem contrast was recalculated and the permutation *P*-value was defined as the proportion of permuted *t* statistics that were as extreme or more extreme than the observed value.

Pairwise genetic differentiation among sampling sites was quantified using *F*_ST_, estimated with the R package *StAMPP* v. 1.6.3 [154]. Confidence intervals for pairwise *F*_ST_ estimates were obtained using 1,000 bootstrap replicates. To test for isolation by distance (IBD), we conducted Mantel tests implemented in the R package *ade4* v. 1.7–22 [155], using linearised *F*_ST_ values [*F*_ST_/(1 − *F*_ST_)] and log-transformed geographic distances between sampling sites.

Population genetic structure was examined using sparse non-negative matrix factorisation (sNMF) [156] implemented in the R package *LEA* v. 3.14 [157]. The number of ancestral populations (K) was evaluated across a range of values from 1 to 19, with ten replicate runs per K. The best-supported K was identified based on the lowest cross-entropy criterion. As an independent and complementary approach, we also inferred population structure using discriminant analysis of principal components (DAPC) implemented in the R package *adegenet* v. 2.1.11 [158]. The optimal number of retained principal components was determined using α-score optimisation (34 PCs for *B. lapidarius*, 32 PCs for *B. terrestris* and 34 PCs for *B. pascuorum*). In addition, the *find.clusters* function in *adegenet* was used to identify the most likely number of genetic clusters by applying successive K-means clustering to PCA-transformed data and selecting the optimal K based on the Bayesian Information Criterion (BIC) scores. All clustering analyses were conducted without prior information on individual group membership.

### SNPs associated with urban environments

To identify SNPs exhibiting allele-frequency divergence associated with urban environments, we applied multiple complementary genome–environment association (GEA) approaches that differ in how genomic–environment relationships are modelled. Specifically, we used a constrained ordination method (redundancy analysis, RDA) and a hierarchical linear mixed model (LMM) incorporating the paired sampling design. In addition, a latent factor mixed model (LFMM) was applied as a complementary analysis to evaluate the robustness of detected associations across alternative modelling frameworks. Although these approaches are not statistically independent because they analyse the same underlying allele-frequency data and urbanisation contrast, they capture genomic–environment associations using different analytical assumptions and statistical structures. For all GEA analyses, missing genotypes were imputed using Beagle v. 5.4 with default parameters [159, 160] and analyses were conducted using population-based allele frequencies. Because realised genotype missingness after filtering was low across all three species (*B. lapidarius*: 2.91%; *B. terrestris:* 3.83%; *B. pascuorum*: 2.01%), only a small proportion of genotype calls required imputation.

To assess the reliability of allele frequency estimates derived from small sample sizes, we performed a subsampling analysis using *B. lapidarius*, the species with the smallest overall sample sizes. We randomly selected 1,000 SNPs and, for each SNP, calculated the absolute deviation between allele frequencies estimated from subsamples of 2–12 individuals and the corresponding full-sample estimate (*n* = 12). We then quantified the proportion of SNPs with deviations below thresholds of 0.01, 0.02, 0.03, and 0.05, corresponding to increasingly relaxed criteria for concordance. This analysis showed that allele frequency estimates based on four individuals showed good concordance with full-sample estimates, with 84% of SNPs exhibiting deviations ≤ 2% (Additional file 2: Fig. S10).

Redundancy analysis (RDA) was used to model multivariate genetic variation as a linear function of environmental predictors. RDAs were performed using the R package *vegan* v2.6–4 [161], with ecosystem type (urban versus rural) included as a constraining variable and geographic coordinates (latitude and longitude) included as covariates. SNPs associated with urban environments were identified based on their loadings on the constrained RDA axis. Candidate loci were defined as those exceeding three median absolute deviations (MADs) from the median loading (Z > 3), providing a robust, distribution-free alternative to conventional standard deviation–based thresholds.

In parallel with RDA, we applied a LMM approach implemented in the R package *nlme* v 3.1–164 [128] to identify SNPs associated with urban environments. For each SNP, population-based allele frequencies were modelled as the response variable, ecosystem type as a fixed effect and site pair as a random intercept to account for the paired sampling design. To control for false discoveries, we performed a permutation-based empirical significance test by randomly swapping ecosystem labels within site pairs 100 times, thereby preserving the paired sampling design while removing the true environmental signal. For each permuted dataset, the full set of LMMs was refitted and the resulting *P*-values were recorded to generate a null distribution. SNPs were retained if their observed *P*-values were more extreme than 99.8% of permutation-derived null associations.

As a complementary robustness analysis, we applied LFMM to test for associations between allele frequencies and urban environments while accounting for potential latent covariance structure among populations [162, 163]. LFMMs were implemented using ridge regression in the *lfmm* R package [162], with the number of latent factors set to K = 1, as suggested by the sNMF analyses indicating weak genome-wide population structure. Population-based allele frequencies were used as response variables, with ecosystem type included as the predictor. Resulting calibrated *P*-values were converted to *q*-values and SNPs with *q* < 0.05 were considered significant. Concordance among LFMM, paired LMM and RDA results was subsequently used to evaluate the robustness of candidate urban-associated loci across alternative analytical frameworks.

Urban-associated SNPs were defined conservatively as those consistently identified across the two primary genome–environment association methods (RDA ∩ LMM). The significance of overlap between methods was assessed using hypergeometric tests implemented with the *phyper* function in R. Using this set of urban-associated SNPs, we conducted a principal component analysis (PCA) to visualise patterns of genetic variation and used the first principal component (PC1) to assess the extent of separation between urban and rural populations. To quantify the consistency of allele-frequency patterns across independent cities, we evaluated, for each urban-associated SNP, the number of urban populations in which the same allele was the minor allele. We then calculated the proportion of SNPs showing concordant minor alleles across one to nine urban populations. To further quantify the extent of parallel genomic divergence across independent urban landscapes, we partitioned allele-frequency variation at candidate urban-associated SNPs into shared ecosystem effects and ecosystem × site pair interaction effects, following a framework similar to that used by Salmón et al. [26]. For each candidate SNP, allele frequencies were modelled as a function of ecosystem (urban versus rural), site pair identity and their interaction using linear models implemented in R. Effect sizes were quantified as η^2^ values calculated from sums of squares obtained from ANOVA models. Shared ecosystem effects therefore represented consistent allele-frequency divergence across replicated urban–rural contrasts, whereas ecosystem × pair interaction effects quantified pair-specific deviations from parallelism.

To investigate broader genomic patterns, we assessed whether urban-associated SNPs were non-randomly distributed across the genome. We first evaluated chromosome-scale patterns by testing the relationship between the number of urban-associated SNPs per chromosome and both total SNP number and gene content per chromosome using linear regression models to account for differences in genomic opportunity among chromosomes. We additionally evaluated the spatial distribution of urban-associated SNPs within chromosomes by dividing each chromosome into non-overlapping 100 kb bins and counting the number of candidate SNPs per bin. A likelihood-ratio G-test was then used to assess whether observed SNP counts deviated from random expectations.

To assess whether urban-associated genomic signatures were consistent with selection acting on standing genetic variation, we examined allele frequencies at urban-associated loci (RDA ∩ LMM) in rural populations. For each candidate SNP, we calculated the mean allele frequency across rural populations and converted this to minor allele frequency (MAF). SNPs with mean rural MAF ≥ 0.1 were considered to be segregating at appreciable frequencies in rural populations and therefore consistent with standing genetic variation [164].

Finally, to explore which components of the urban environment were associated with allele-frequency variation at candidate loci (RDA ∩ LMM), we modelled population-level allele frequencies of urban-associated SNPs against land-surface temperature, road density, PM_2_._5_ and NO_2_, with site pair included as a random intercept. Road density was quantified as the total length of roads within a 1 km radius of each sampling site using road-network shapefiles obtained from Geofabrik GmbH. All environmental predictors were standardised prior to analysis. Because road density and land-surface temperature were strongly correlated and showed elevated variance inflation factors (VIF > 3) when included simultaneously, they were evaluated in separate models, with PM_2_._5_ and NO_2_ retained as predictors in both. Thus, the two models evaluated land-surface temperature + PM_2_._5_ + NO_2_ and road density + PM_2_._5_ + NO_2_, respectively. *P*-values were corrected across candidate SNPs separately for each predictor and species using the Benjamini–Hochberg FDR procedure, with FDR-adjusted *P* < 0.05 considered significant.

### Selective sweep analysis

To detect genomic regions showing signatures of selective sweeps between urban and rural bumble bee populations, we used the cross-population nSL statistic (XP-nSL), implemented in selscan v. 2.0.2 [165]. XP-nSL is a haplotype-based statistic designed to detect positive selection by comparing haplotype homozygosity between populations, and it has power to detect both ongoing and recently completed selective sweeps, including hard sweeps arising from de novo mutations and soft sweeps acting on standing genetic variation [166]. Specifically, XP-nSL identifies genomic regions in which haplotypes in a focal population (urban) exhibit extended homozygosity relative to a reference population (rural).

Analyses were conducted separately for each of the nine paired urban–rural population comparisons. To account for variation in SNP density and background haplotype structure, raw XP-nSL scores were normalised in non-overlapping 50 kb windows using the *norm* program implemented in *selscan* v. 2.0.2 [165]. Windows were considered to show signatures of selection in urban populations if their normalised XP-nSL values were positive and exceeded three median absolute deviations above the median (Z > 3).

To assess the consistency of selective sweep signals across independent urban–rural comparisons, we implemented a resampling-based null model. For each urban–rural pair, we randomly resampled (with replacement) *n* windows (*n* = number of outlier windows detected for that pair) from the full set of genomic windows. For each iteration, we quantified the number of windows shared among two to nine population pairs and repeated this procedure 100,000 times to generate empirical null distributions of expected overlap. The mean and 95% confidence intervals (CIs) of the expected number of shared windows were then compared with the observed overlap in the empirical data. Across all species, the expected number of windows shared among four or more urban–rural pairs under random sampling was effectively zero. Accordingly, genomic regions detected in at least four of nine urban–rural comparisons were retained as recurrent candidate sweep regions consistently associated with urban environments. These candidate regions were visualised using Manhattan plots of normalised XP-nSL scores calculated from comparisons between all urban individuals and all rural individuals. To account for uncertainty in window boundaries, adjacent XP-nSL outlier windows separated by two or fewer windows (± 100 kb) were merged into extended candidate regions.

To integrate selective sweep results with GEA analyses, we compared XP-nSL outlier regions with SNPs identified by RDA and LMM. SNPs located within 10 kb upstream or downstream of the boundaries of XP-nSL outlier regions were considered overlapping. This buffer distance was chosen to account for local linkage disequilibrium and minor positional uncertainty. Genomic regions supported by both selection scans and GEA analyses were also considered strong candidates for urban-associated genomic divergence, defined as SNPs jointly identified by RDA and LMM (RDA ∩ LMM), together with additional SNPs identified by either GEA approach that overlapped XP-nSL selective sweep regions (XP-nSL ∩ (RDA ∪ LMM)). To test whether overlap exceeded random expectations, we calculated the fraction of the genome covered by XP-nSL outlier regions and used a one-sided binomial test to assess whether the number of XP-nSL regions overlapping urban-associated SNPs was greater than expected under a uniform genomic distribution.

To evaluate whether linked selection and recombination rate variation influenced the distribution of XP-nSL outliers, we quantified linkage disequilibrium (LD) and intronic GC content in 50 kb windows and examined their relationship with XP-nSL scores. Linked selection is expected to reduce genetic diversity disproportionately in genomic regions of low recombination, where linkage is strong. Intronic GC content can serve as a proxy for long-term recombination rate because it reflects the cumulative effects of mutation bias, gene conversion and selection over evolutionary timescales [167].

If linked selection associated with recombination-rate variation strongly influences patterns of genomic divergence, we expect XP-nSL scores to be systematically associated with estimates of LD and intronic GC content. LD was estimated using PLINK v.1.9 (--r2 --ld-window-r2 0 --ld-window-kb 50) for SNP pairs up to 50 kb apart, using all urban individuals. LD estimates were averaged in non-overlapping 50 kb windows using the R package WindowScanR v.0.1 (https://github.com/tavareshugo/WindowScanR). Intronic GC content was estimated for each species using annotated genomes (*B. terrestris*: iyBomTerr1.2 [132, 133]; *B. pascuorum*: iyBomPasc1.1 [134, 135]). For *B. lapidarius*, which lacked a published annotation, we annotated the reference genome (iyBomLapi1.1 [130, 131]) using BRAKER2 v.2.1.4 [168], GALBA v.1.0.11 [169] and Funannotate v.1.8.17 [170], all with default parameters and with *B. terrestris* as the closest reference species. Gene models were combined and filtered using EvidenceModeler v. 2.1.0 [171], yielding a high-confidence annotation with 98.1% completeness as assessed by BUSCO v. 5 [172]. Publicly available annotations for *B. terrestris* (iyBomTerr1.2 [132, 133]; annotation release 103; GCF_910591885.1) and *B. pascuorum* (iyBomPasc1.1 [134, 135]; GCF_905332965.1-RS_2023_11; GCF_905332965.1) also showed similarly high BUSCO completeness (99.1% and 99.3%, respectively), indicating broadly comparable annotation quality across species.

Intronic coordinates were extracted using the R packages GenomicFeatures v.1.54.4 [173] and rtracklayer v.1.62.0 [174], and GC content was calculated using BEDtools v.2.28 [175]. GC content values were averaged in 50 kb windows using the R package WindowScanR. For each chromosome and species, we calculated Pearson correlations between XP-nSL scores, LD and intronic GC content using the *cor.test* function in R. To assess genome-wide significance, correlation coefficients were aggregated across chromosomes using Fisher’s *z*-transformation, and the mean *z* value was tested against zero.

### Functional characterisation of candidate loci under selection

We functionally annotated urban-associated SNPs using SnpEff v.5.2 [176], which predicts the genomic context of each variant (intronic, intergenic, upstream, downstream, untranslated regions (UTRs), transcript-associated non-coding regions and protein-coding exons) and their predicted functional consequences (e.g. synonymous and missense substitutions). Because individual SNPs can be annotated to multiple genomic features across alternative transcripts, annotations were summarised using a hierarchical priority scheme, assigning each SNP a single primary genomic category to avoid redundant classification. For each species, we annotated the core set of SNPs jointly identified by RDA and LMM (RDA ∩ LMM) and additionally included SNPs identified by either GEA approach that overlapped XP-nSL selective sweep regions (12 in *B. lapidarius*, 32 in *B. terrestris* and 313 in *B. pascuorum*).

Additionally, we identified urban-associated genes for each species by mapping candidate SNPs to nearby genes using BEDtools v.2.28 [175]. Candidate SNPs comprised (i) the core set jointly supported by genome–environment association analyses (RDA ∩ LMM) and (ii) SNPs detected by either GEA approach (RDA or LMM) that overlapped XP-nSL outlier regions. Genes were defined as putatively associated with urbanisation if they occurred within ± 10 kb of a candidate SNP. This window was chosen to account for the potential contribution of nearby regulatory variation while remaining biologically conservative given the relatively high recombination rates and expected short-range linkage disequilibrium in bumble bees [177]. For *B. terrestris* and *B. pascuorum*, we used the annotated reference genomes iyBomTerr1.2 [132, 133] (annotation release 103; “GCF_910591885.1”) and iyBomPasc1.1 [134, 135] (GCF_905332965.1-RS_2023_11; “GCF_905332965.1”), respectively. For *B. lapidarius*, we used our annotated reference genome iyBomLapi1.1 [130, 131] (GCA_964186655.1). Gene ontology enrichment tests were conducted using the R package topGO v2.54.0 [178, 179]. Species-specific annotation data were used as the background gene set. For *B. lapidarius*, we obtained functional annotations using InterProScan implemented in OmicsBox 3.4 [180]. The final annotated gene set of *B. lapidarius* consisted of 13,516 genes with 5,569 associated GO terms, as detailed in the supplementary data provided on Figshare (blapidarius.cds and blapidarius_wego) [181]. We used the topology-aware *elim* algorithm because Gene Ontology categories are hierarchically structured and statistically non-independent due to extensive overlap among parent and child terms. The *elim* algorithm was specifically developed to reduce local dependency and redundancy within the GO graph by iteratively removing genes mapped to significant child terms from higher-order ancestral categories, thereby improving biological interpretability and reducing false-positive enrichment caused by GO topology [178, 179]. A minimum node size of five annotated genes was used, following the default settings and recommendations of the topGO documentation, thereby excluding GO terms with extremely low annotation support [178, 179]. GO terms with *P* < 0.05, under the topology-aware *elim* framework, were considered enriched. For visualisation, we ranked terms by *P*-value and retained the 10 most significant enriched GO terms (*P* < 0.05) within each ontology (biological process, molecular function and cellular component). We additionally performed complementary enrichment analyses using the classic topGO algorithm, with *P*-values adjusted for multiple testing using the Benjamini–Hochberg false discovery rate (FDR) procedure, and provide these results in the Supplementary Material, while noting that conventional multiple-testing correction alone does not explicitly account for the hierarchical dependency structure among GO categories. To assess concordance between the two enrichment approaches, we tested the overlap in significant GO terms between the *elim* and FDR-corrected analyses at the primary ± 10 kb SNP-to-gene assignment window using one-sided hypergeometric tests. To further evaluate the sensitivity of functional enrichment patterns to SNP-to-gene assignment distance, we repeated GO enrichment analyses using conservative within-gene assignments (0 kb) and an intermediate ± 5 kb proximity window, in addition to the primary ± 10 kb window and compared overlap among enriched GO-term sets across assignment approaches under both the *elim* and FDR-corrected analyses. Significance of overlap among enriched GO-term sets was assessed using one-sided hypergeometric tests, with *P*-values corrected across comparisons using the Benjamini–Hochberg procedure.

### Parallels across species

To examine convergence in genomic responses to urban landscapes across species, we tested for shared urban-associated genes among bumble bees. Candidate gene sets were defined independently for each species as genes located within ± 10 kb of urbanisation-associated loci, comprising SNPs jointly identified by RDA and LMM (RDA ∩ LMM) together with additional SNPs identified by either GEA approach (RDA or LMM) that overlapped XP-nSL selective sweep regions. Orthologous relationships among genes were inferred using OrthoFinder [182], allowing candidate genes to be matched across species based on shared orthogroups. Pairwise overlap in orthologous candidate genes between species was assessed using one-sided hypergeometric tests implemented with the *phyper* function in R. For each comparison, the background universe comprised all genes with orthology information available between the species pair, ensuring that overlap was evaluated within a shared, orthology-defined gene set. To assess the sensitivity of cross-species gene overlap to SNP-to-gene assignment distance, pairwise orthologous gene overlap was additionally evaluated using candidate gene sets defined at 0 kb and ± 5 kb, alongside the primary ± 10 kb assignment. To evaluate convergence across all three species simultaneously, we quantified the number of candidate genes shared among *B. lapidarius*, *B. terrestris* and *B. pascuorum*. Because three-way overlap cannot be evaluated using a single hypergeometric test, significance was assessed using a permutation test. The background universe comprised genes with orthology information available across all three species. In each of 10,000 permutations, we randomly sampled from this universe the same number of candidate genes as observed in each species-specific set and recalculated the size of the three-way intersection. Statistical significance was assessed using a one-sided permutation test, defined as the proportion of permutations yielding an overlap equal to or greater than the observed overlap.

Gene Ontology (GO) enrichment analyses for genes shared across species were conducted using the R package topGO v2.54.0 [179]. Annotation data from the most extensively characterised *Bombus* species, *B. terrestris*, were used as the background set for cross-species GO enrichment analyses because *B. terrestris* serves as a common reference framework for *Bombus* functional annotation resources. We used the conservative *elim* algorithm in topGO, which reduces false positives by down-weighting broad parent terms once significant child terms are identified. A minimum node size of five annotated genes was used, following the default recommendations of the topGO documentation, thereby excluding GO terms with extremely low annotation support [179]. GO terms with *elim P* < 0.05 were considered enriched under the topology-aware framework. For visualisation, we ranked terms by *P*-value and retained the 10 most significant enriched GO terms (*P* < 0.05) within each ontology (biological process, molecular function and cellular component).

To assess functional convergence in genomic responses to urban environments across species, we quantified semantic similarity among enriched GO terms using the R package *GOSemSim* [183, 184]. Semantic similarity was calculated using the Wang method, which incorporates the topology and hierarchical structure of the GO graph. Analyses included GO terms from all three ontologies—biological process, molecular function, and cellular component—to capture convergence across complementary functional dimensions. Because annotation completeness differed among species, GO background universes were defined separately for each species pair as the intersection of all GO terms annotated in both species under comparison, thereby restricting analyses to functionally comparable terms. For each species pair, semantic similarity between enriched GO-term sets was summarised using the best-match average (BMA) across all GO terms within each ontology, and ontology-specific similarity scores were averaged to obtain a combined pairwise similarity metric. Statistical significance was evaluated using permutation tests. For each permutation, GO terms were randomly sampled without replacement from the corresponding pairwise GO universe, matching the observed number of enriched terms in each species, and semantic similarity was recalculated using the same procedure. This was repeated 10,000 times to generate null distributions for each pairwise comparison. One-sided permutation *P*-values were calculated as the proportion of permuted similarity scores equal to or greater than the observed value.

## Supplementary Information

Additional file 1: Supplementary tables. Tables S1–S39, including genetic diversity, population differentiation, genomic associations, selective sweep regions, functional annotation, sampling information, sequencing statistics and relatedness analyses.

Additional file 2: Supplementary figures. Figs. S1–S10, showing demographic inference, isolation by distance, population structure, overlap among association methods, structural variation, functional enrichment and robustness analyses.

## Data Availability

The raw whole-genome sequencing data generated in this study have been deposited in the NCBI Sequence Read Archive (SRA) under BioProject accession PRJNA1117648 [185]. The variant call format (VCF) files, *Bombus lapidarius* genome annotation files and scripts developed for data analysis are available on figshare (https://doi.org/10.6084/m9.figshare.26303974) [181]. The analysis scripts are also publicly available on GitHub (https://github.com/panastheod/Parallel_urban_bumble_bee_genomics) [186] and are released under the MIT License. The publicly available reference genome assemblies used in this study were *Bombus lapidarius* (iyBomLapi1.1; GenBank accession GCA_964186655.1) [130, 131], *Bombus terrestris* (iyBomTerr1.2; RefSeq accession GCF_910591885.1) [132, 133] and *Bombus pascuorum* (iyBomPasc1.1; GenBank accession GCA_905332965.1; corresponding RefSeq accession GCF_905332965.1) [134, 135]. The reference genome datasets and their associated genome publications are cited separately to acknowledge the original data providers. All software tools and R packages used in the genomic analyses are described in the Methods section.

## References

[1] Charmantier A, Burkhard T, Gervais L, Perrier C, Schulte-Hostedde AI, Thompson MJ. How does urbanization affect natural selection? Funct Ecol. 2024;38:2522–36. 10.1111/1365-2435.14667.

[2] Johnson MTJ, Munshi-South J. Evolution of life in urban environments. Science. 2017;358:eaam8327. 10.1126/science.aam8327. 29097520

[3] Li G, Fang C, Li Y, Wang Z, Sun S, He S, et al. Global impacts of future urban expansion on terrestrial vertebrate diversity. Nat Commun. 2022;13:1628. 10.1038/s41467-022-29324-2. PMC8956596 35338145

[4] Huang K, Li X, Liu X, Seto KC. Projecting global urban land expansion and heat island intensification through 2050. Environ Res Lett. 2019;14:114037. 10.1088/1748-9326/ab4b71.

[5] United Nations, Department of Economic and Social Affairs, Population Division. World population prospects: the 2018 revision, methodology of the United Nations population estimates and projections. New York: United Nations; 2019.

[6] McDonnell MJ, Hahs AK. Comparative ecology of cities and towns: Past, present and future. In: ecology of cities and towns: a comparative approach. 2009. 10.1017/cbo9780511609763.006.

[7] Santangelo JS, Ness RW, Cohan B, Fitzpatrick CR, Innes SG, Koch S, et al. Global urban environmental change drives adaptation in white clover. Science. 2022;375:1275–81. 10.1126/science.abk0989. 35298255

[8] Rivkin LR, Santangelo JS, Alberti M, Aronson MFJ, de Keyzer CW, Diamond SE, et al. A roadmap for urban evolutionary ecology. Evol Appl. 2019;12:384–98. 10.1111/eva.12734. PMC6383741 30828362

[9] Smith TB, Bernatchez L. Evolutionary change in human-altered environments. Mol Ecol. 2008;17:1–8. 10.1111/j.1365-294x.2007.03607.x. 18173497

[10] Donihue CM, Lambert MR. Adaptive evolution in urban ecosystems. Ambio. 2015;44:194–203. 10.1007/s13280-014-0547-2. PMC4357625 25056615

[11] Szulkin M, Munshi-South J, Charmantier A. Urban evolutionary biology. Oxford University Press; 2020. 10.1093/oso/9780198836841.001.0001.

[12] Cheptou P-O, Carrue O, Rouifed S, Cantarel A. Rapid evolution of seed dispersal in an urban environment in the weed Crepis sancta. Proc Natl Acad Sci. 2008;105:3796–9. 10.1073/pnas.0708446105. PMC2268839 18316722

[13] Diamond SE, Cayton H, Wepprich T, Jenkins CN, Dunn RR, Haddad NM, et al. Unexpected phenological responses of butterflies to the interaction of urbanization and geographic temperature. Ecology. 2014;95:2613–21. 10.1890/13-1848.1.

[14] Kearney MR, Briscoe NJ, Karoly DJ, Porter WP, Norgate M, Sunnucks P, et al. Early emergence in a butterfly causally linked to anthropogenic warming. Biol Let. 2010;6:674–7. 10.1098/rsbl.2010.0053. PMC2936131 20236964

[15] Angilletta MJ, Wilson RS, Niehaus AC, Sears MW, Navas CA, Ribeiro PL. Urban Physiology: City Ants Possess High Heat Tolerance. PLoS ONE. 2007;2:e258. 10.1371/journal.pone.0000258. PMC1797824 17327918

[16] Theodorou P, Baltz LM, Paxton RJ, Soro A. Urbanization is associated with shifts in bumblebee body size, with cascading effects on pollination. Evol Appl. 2021;14:53–68. 10.1111/eva.13087. PMC7819558 33519956

[17] Campbell-Staton SC, Winchell KM, Rochette NC, Fredette J, Maayan I, Schweizer RM, et al. Parallel selection on thermal physiology facilitates repeated adaptation of city lizards to urban heat islands. Nature Ecology & Evolution. 2020;4:652–8. 10.1038/s41559-020-1131-8. 32152530

[18] Tomotani BM, Couweleers M, ten Brinke B, Walboom A, van Oers K, Visser ME. Size in the city: morphological differences between city and forest great tits have a genetic basis. Evolution Letters. 2025;9:317–23. 10.1093/evlett/qraf006. PMC12137048 40487867

[19] Woudstra Y, Kraaiveld R, Jorritsma A, Vijverberg K, Ivanovic S, Erkens R, et al. Some like it hot: adaptation to the urban heat island in common dandelion. Evolution Letters. 2024;8:881–92. 10.1093/evlett/qrae040. PMC11637554 39677581

[20] Babik W, Dudek K, Marszałek M, Palomar G, Antunes B, Sniegula S. The genomic response to urbanization in the damselfly Ischnura elegans. Evol Appl. 2023;16:1805–18. 10.1111/eva.13603. PMC10681423 38029064

[21] Harris SE, Munshi-South J, Obergfell C, O’Neill R, Shochat E, Warren P, et al. Signatures of Rapid Evolution in Urban and Rural Transcriptomes of White-Footed Mice (Peromyscus leucopus) in the New York Metropolitan Area. PLoS ONE. 2013;8:e74938. 10.1371/journal.pone.0074938. PMC3756007 24015321

[22] Harris SE, Munshi-South J. Signatures of positive selection and local adaptation to urbanization in white-footed mice (Peromyscus leucopus). Mol Ecol. 2017;26:6336–50. 10.1111/mec.14369. PMC5716853 28980357

[23] Mueller JC, Partecke J, Hatchwell BJ, Gaston KJ, Evans KL. Candidate gene polymorphisms for behavioural adaptations during urbanization in blackbirds. Mol Ecol. 2013;22:3629–37. 10.1111/mec.12288. 23495914

[24] Mueller JC, Carrete M, Boerno S, Kuhl H, Tella JL, Kempenaers B. Genes acting in synapses and neuron projections are early targets of selection during urban colonization. Mol Ecol. 2020;29:3403–12. 10.1111/mec.15451. 32310323

[25] Perrier C, Lozano del Campo A, Szulkin M, Demeyrier V, Gregoire A, Charmantier A. Great tits and the city: distribution of genomic diversity and gene–environment associations along an urbanization gradient. Evol Appl. 2018;11:593–613. 10.1111/eva.12580. PMC5979639 29875805

[26] Salmón P, Jacobs A, Ahrén D, Biard C, Dingemanse NJ, Dominoni DM, et al. Continent-wide genomic signatures of adaptation to urbanisation in a songbird across Europe. Nat Commun. 2021;12:2983. 10.1038/s41467-021-23027-w. PMC8137928 34016968

[27] Theodorou P, Radzevičiūtė R, Kahnt B, Soro A, Grosse I, Paxton RJ. Genome-wide single nucleotide polymorphism scan suggests adaptation to urbanization in an important pollinator, the red-tailed bumblebee (Bombus lapidarius L.). Proceed Royal Soc B: Biol Sci. 2018;285:20172806. 10.1098/rspb.2017.2806. PMC5936727 29669900

[28] Thompson KA, Renaudin M, Johnson MTJ. Urbanization drives the evolution of parallel clines in plant populations. Proceedings of the Royal Society B: Biological Sciences. 2016;283:20162180. 10.1098/rspb.2016.2180. PMC5204167 28003451

[29] Kivelä SM, Lee KM, Adamidis GC, Bonelli S, Hendrickx F, Huemer P, et al. Population history shapes urban evolutionary dynamics: distinct genetic structure across urban and rural Europe in two lepidopterans. Proc Biol Sci. 2026;293:20252434. 10.1098/rspb.2025.2434. 41558783

[30] Savolainen O, Lascoux M, Merilä J. Ecological genomics of local adaptation. Nat Rev Genet. 2013;14:807–20. 10.1038/nrg3522. 24136507

[31] Shafer ABA, Wolf JBW, Alves PC, Bergström L, Bruford MW, Brännström I, et al. Genomics and the challenging translation into conservation practice. Trends Ecol Evol. 2015;30:78–87. 10.1016/j.tree.2014.11.009. 25534246

[32] Barghi N, Hermisson J, Schlötterer C. Polygenic adaptation: a unifying framework to understand positive selection. Nat Rev Genet. 2020;21:769–81. 10.1038/s41576-020-0250-z. 32601318

[33] Chaturvedi N, Chatterjee P. Evolutionary Adaptation in Heterogeneous and Changing Environments. Evolution. 2025;79:119–33. 10.1093/evolut/qpae144. 39382343

[34] Kreiner JM. The genetic architecture and spatiotemporal dynamics of adaptation across human-modified landscapes. New Phytol. 2026;249:744–50. 10.1111/nph.70520. PMC12712421 40919705

[35] Campagna L, Toews DPL. The genomics of adaptation in birds. Curr Biol. 2022;32:R1173–86. 10.1016/j.cub.2022.07.076. 36283387

[36] Wray GA. The evolutionary significance of cis-regulatory mutations. Nat Rev Genet. 2007;8:206–16. 10.1038/nrg2063. 17304246

[37] Wilson EO. The little things that run the world* (the importance and conservation of invertebrates). Conserv Biol. 1987;1:344–6. 10.1111/j.1523-1739.1987.tb00055.x.

[38] Price PW. Insect studies in the advancement of science: From Darwin and Wallace to the present. Am Entomol. 2003;49:164–73. 10.1093/ae/49.3.164.

[39] Woodard SH, Lozier JD, Goulson D, Williams PH, Strange JP, Jha S. Molecular tools and bumble bees: revealing hidden details of ecology and evolution in a model system. Mol Ecol. 2015;24:2916–36. 10.1111/mec.13198. 25865395

[40] Webster MT, Beaurepaire A, Neumann P, Stolle E. Population genomics for insect conservation. Annual Review of Animal Biosciences. 2023;11:115–40. 10.1146/annurev-animal-122221-075025. 36375448

[41] Huml JV, Ellis JS, Rustage S, Brown MJF, Billington R, Knight ME. The tragedy of the common? A comparative population genomic study of two bumblebee species. Insect Conservation and Diversity. 2023;16:335–54. 10.1111/icad.12626.

[42] Heraghty SD, Jackson JM, Lozier JD. Whole genome analyses reveal weak signatures of population structure and environmentally associated local adaptation in an important North American pollinator, the bumble bee Bombus vosnesenskii. Mol Ecol. 2023;32:5479–97. 10.1111/mec.17125. 37702957

[43] Sousa P, Theodorou P, Martins AC, Aguiar AJC, Shibata A, Kahnt B. Integrating population genomics and historical occurrence data to assess the impact of land-use changes on Centris oil bees in the Brazilian Cerrado. Mol Ecol. 2025;34:e70153. 10.1111/mec.70153. 41117429

[44] Baltz LM, Gardein H, Greil H, Paxton RJ, Theodorou P. Different Paths, Similar Pressures: Divergent Drivers of Genetic Diversity Despite Convergent Genomic Signatures of Selection in Response to Urban Intensity in Two Oligolectic Bee Species. Mol Ecol. 2026;35:e70370. 10.1111/mec.70370. PMC13156532 42104688

[45] Baldock KCR, Goddard MA, Hicks DM, Kunin WE, Mitschunas N, Osgathorpe LM, et al. Where is the UK’s pollinator biodiversity? The importance of urban areas for flower-visiting insects. Proceedings of the Royal Society B: Biological Sciences. 2015;282:20142849. 10.1098/rspb.2014.2849. PMC4345454 25673686

[46] Bates AJ, Sadler JP, Fairbrass AJ, Falk SJ, Hale JD, Matthews TJ. Changing bee and hoverfly pollinator assemblages along an urban-rural gradient. PLoS ONE. 2011;6:e23459. 10.1371/journal.pone.0023459. PMC3155562 21858128

[47] Deguines N, Julliard R, de Flores M, Fontaine C. Functional homogenization of flower visitor communities with urbanization. Ecol Evol. 2016;6:1967–76. 10.1002/ece3.2009. PMC4767875 27066219

[48] Fortel L, Henry M, Guilbaud L, Guirao AL, Kuhlmann M, Mouret H, et al. Decreasing abundance, increasing diversity and changing structure of the wild bee community (Hymenoptera: Anthophila) along an urbanization gradient. PLoS ONE. 2014;9:e104679. 10.1371/journal.pone.0104679. PMC4131891 25118722

[49] Hofmann MM, Zohner CM, Renner SS. Narrow habitat breadth and late-summer emergence increases extinction vulnerability in Central European bees. Proceedings of the Royal Society B: Biological Sciences. 2019;286:20190316. 10.1098/rspb.2019.0316. PMC6458332 30836868

[50] Liang H, He Y-D, Theodorou P, Yang C-F. The effects of urbanization on pollinators and pollination: a meta-analysis. Ecol Lett. 2023;26:1629–42. 10.1111/ele.14277. 37345567

[51] Samuelson AE, Gill RJ, Brown MJF, Leadbeater E. Lower bumblebee colony reproductive success in agricultural compared with urban environments. Proceedings of the Royal Society B: Biological Sciences. 2018;285:20180807. 10.1098/rspb.2018.0807. PMC6030522 30051852

[52] Theodorou P, Radzevičiūtė R, Settele J, Schweiger O, Murray TE, Paxton RJ. Pollination services enhanced with urbanization despite increasing pollinator parasitism. Proceedings of the Royal Society B: Biological Sciences. 2016;283:20160561. 10.1098/rspb.2016.0561. PMC4936033 27335419

[53] Theodorou P, Albig K, Radzevičiūtė R, Settele J, Schweiger O, Murray TE, et al. The structure of flower visitor networks in relation to pollination across an agricultural to urban gradient. Funct Ecol. 2017;31:838–47. 10.1111/1365-2435.12803.

[54] Theodorou P, Radzevičiūtė R, Lentendu G, Kahnt B, Husemann M, Bleidorn C, et al. Urban areas as hotspots for bees and pollination but not a panacea for all insects. Nat Commun. 2020;11:576. 10.1038/s41467-020-14496-6. PMC6989530 31996690

[55] Theodorou P, Kühn O, Baltz LM, Wild C, Rasti SL, Bucksch CR, et al. Bumble bee colony health and performance vary widely across the urban ecosystem. J Anim Ecol. 2022;91:2135–48. 10.1111/1365-2656.13797. 36002939

[56] Verboven HAF, Aertsen W, Brys R, Hermy M. Pollination and seed set of an obligatory outcrossing plant in an urban–peri-urban gradient. Perspectives in Plant Ecology, Evolution and Systematics. 2014;16:121–31. 10.1016/j.ppees.2014.03.002.

[57] Ballare KM, Jha S. Genetic structure across urban and agricultural landscapes reveals evidence of resource specialization and philopatry in the eastern carpenter bee. Xylocopa virginica L. Evolutionary Applications. 2021;14:136–49. 10.1111/eva.13078. PMC7819568 33519961

[58] Chau KD, Samad-zada F, Kelemen EP, Rehan SM. Integrative population genetics and metagenomics reveals urbanization increases pathogen loads and decreases connectivity in a wild bee. Glob Change Biol. 2023;29:4193–211. 10.1111/gcb.16757. 37173859

[59] Jha S. Contemporary human-altered landscapes and oceanic barriers reduce bumble bee gene flow. Mol Ecol. 2015;24:993–1006. 10.1111/mec.13090. 25626470

[60] Jha S, Kremen C. Urban land use limits regional bumble bee gene flow. Mol Ecol. 2013;22:2483–95. 10.1111/mec.12275. 23495763

[61] Goulson D. Bumblebees: behaviour, ecology, and conservation. Oxford: Oxford University Press; 2010.

[62] Banaszak-Cibicka W, Żmihorski M. Wild bees along an urban gradient: winners and losers. J Insect Conserv. 2012;16:331–43. 10.1007/s10841-011-9419-2.

[63] Goulson D, Darvill B. Niche overlap and diet breadth in bumblebees; are rare species more specialized in their choice of flowers? Apidologie. 2004;35:55–63. 10.1051/apido:2003062.

[64] Huang J, An J, Wu J, Williams PH. Extreme food-plant specialisation in Megabombus bumblebees as a product of long tongues combined with short nesting seasons. PLoS ONE. 2015;10:e0132358. 10.1371/journal.pone.0132358. PMC4534414 26267382

[65] Yang L, Qian F, Song D-X, Zheng K-J. Research on Urban Heat-Island Effect. Procedia Engineering. 2016;169:11–8. 10.1016/j.proeng.2016.10.002.

[66] Baldock KCR. Opportunities and threats for pollinator conservation in global towns and cities. Current Opinion in Insect Science. 2020;38:63–71. 10.1016/j.cois.2020.01.006. 32126514

[67] Jackson JM, Pimsler ML, Oyen KJ, Koch-Uhuad JB, Herndon JD, Strange JP, et al. Distance, elevation and environment as drivers of diversity and divergence in bumble bees across latitude and altitude. Mol Ecol. 2018;27:2926–42. 10.1111/mec.14735. 29862596

[68] Jackson JM, Pimsler ML, Oyen KJ, Strange JP, Dillon ME, Lozier JD. Local adaptation across a complex bioclimatic landscape in two montane bumble bee species. Mol Ecol. 2020;29:920–39. 10.1111/mec.15376. 32031739

[69] Kraus FB, Wolf S, Moritz RFA. Male flight distance and population substructure in the bumblebee *Bombus terrestris*. J Anim Ecol. 2009;78:247–52. 10.1111/j.1365-2656.2008.01479.x. 19120605

[70] Lepais O, Darvill B, O’connor S, Osborne JL, Sanderson RA, Cussans J, et al. Estimation of bumblebee queen dispersal distances using sibship reconstruction method. Mol Ecol. 2010;19:819–31. 10.1111/j.1365-294x.2009.04500.x. 20089127

[71] Darvill B, Knight ME, Goulson D. Use of genetic markers to quantify bumblebee foraging range and nest density. Oikos. 2004;107:471–8. 10.1111/j.0030-1299.2004.13510.x.

[72] Geib JC, Strange JP, Galen C. Bumble bee nest abundance, foraging distance, and host-plant reproduction: implications for management and conservation. Ecol Appl. 2015;25:768–78. 10.1890/14-0151.1. 26214921

[73] Knight ME, Martin AP, Bishop S, Osborne JL, Hale RJ, Sanderson RA, et al. An interspecific comparison of foraging range and nest density of four bumblebee (Bombus) species. Mol Ecol. 2005;14:1811–20. 10.1111/j.1365-294x.2005.02540.x. 15836652

[74] Capblancq T, Forester BR. Redundancy analysis: A Swiss Army Knife for landscape genomics. Methods Ecol Evol. 2021;12:2298–309. 10.1111/2041-210x.13722.

[75] Clark JD, Benham PM, Maldonado JE, Luther DA, Lim HC. Maintenance of local adaptation despite gene flow in a coastal songbird. Evolution. 2022;76:1481–94. 10.1111/evo.14538. PMC9545442 35700208

[76] Henriques D, Wallberg A, Chávez-Galarza J, Johnston JS, Webster MT, Pinto MA. Whole genome SNP-associated signatures of local adaptation in honeybees of the Iberian Peninsula. Sci Rep. 2018;8:11145. 10.1038/s41598-018-29469-5. PMC6057950 30042407

[77] Kardum Hjort C, Paris JR, Smith HG, Dudaniec RY. Selection despite low genetic diversity and high gene flow in a rapid island invasion of the bumblebee Bombus terrestris. Molecular Ecology. 2024;33:e17212. 10.1111/mec.17212. 37990959

[78] Pujolar JM, Jacobsen MW, Als TD, Frydenberg J, Munch K, Jónsson B, et al. Genome-wide single-generation signatures of local selection in the panmictic European eel. Mol Ecol. 2014;23:2514–28. 10.1111/mec.12753. 24750353

[79] Tigano A, Friesen VL. Genomics of local adaptation with gene flow. Mol Ecol. 2016;25:2144–64. 10.1111/mec.13606. 26946320

[80] Faria R, Johannesson K, Butlin RK, Westram AM. Evolving Inversions. Trends Ecol Evol. 2019;34:239–48. 10.1016/j.tree.2018.12.005. 30691998

[81] Charlesworth B, Barton NH. The Spread of an Inversion with Migration and Selection. Genetics. 2018;208:377–82. 10.1534/genetics.117.300426. PMC5753870 29158424

[82] Kirkpatrick M, Barton N. Chromosome Inversions, Local Adaptation and Speciation. Genetics. 2006;173:419–34. 10.1534/genetics.105.047985. PMC1461441 16204214

[83] Schaal SM, Haller BC, Lotterhos KE. Inversion invasions: when the genetic basis of local adaptation is concentrated within inversions in the face of gene flow. Philosophical Transactions of the Royal Society B: Biological Sciences. 2022;377:20210200. 10.1098/rstb.2021.0200. PMC9189506 35694752

[84] Nowling RJ, Manke KR, Emrich SJ. Detecting inversions with PCA in the presence of population structure. PLoS ONE. 2020;15:e0240429. 10.1371/journal.pone.0240429. PMC7595445 33119626

[85] Nowling RJ, Fallas-Moya F, Sadovnik A, Emrich S, Aleck M, Leskiewicz D, et al. Fast, low-memory detection and localization of large, polymorphic inversions from SNPs. PeerJ. 2022;10:e12831. 10.7717/peerj.12831. PMC8784018 35116204

[86] Hermisson J. Who believes in whole-genome scans for selection? Heredity. 2009;103:283–4. 10.1038/hdy.2009.101. 19654610

[87] Kidner J, Theodorou P, Engler JO, Taubert M, Husemann M. A brief history and popularity of methods and tools used to estimate micro-evolutionary forces. Ecol Evol. 2021;11:13723–43. 10.1002/ece3.8076. PMC8525119 34707813

[88] Vitti JJ, Grossman SR, Sabeti PC. Detecting natural selection in genomic data. Annu Rev Genet. 2013;47:97–120. 10.1146/annurev-genet-111212-133526. 24274750

[89] Hancock AM, Alkorta-Aranburu G, Witonsky DB, Di Rienzo A. Adaptations to new environments in humans: The role of subtle allele frequency shifts. Philosophical Transactions of the Royal Society B: Biological Sciences. 2010;365:2459–68. 10.1098/rstb.2010.0032. PMC2935101 20643735

[90] Pritchard JK, Pickrell JK, Coop G. The Genetics of Human Adaptation: Hard Sweeps, Soft Sweeps, and Polygenic Adaptation. Curr Biol. 2010;20:R208–15. 10.1016/j.cub.2009.11.055. PMC2994553 20178769

[91] Pritchard JK, Di Rienzo A. Adaptation—not by sweeps alone. Nat Rev Genet. 2010;11:665–7. 10.1038/nrg2880. PMC4652788 20838407

[92] Baesjou J-P, Wellenreuther M. Genomic signatures of domestication selection in the Australasian snapper (*Chrysophrys auratus*). Genes (Basel). 2021;12:1737. 10.3390/genes12111737. PMC8623400 34828341

[93] Andolfatto P. Adaptive evolution of non-coding DNA in Drosophila. Nature. 2005;437:1149–52. 10.1038/nature04107. 16237443

[94] Božičević V, Hutter S, Stephan W, Wollstein A. Population genetic evidence for cold adaptation in European Drosophila melanogaster populations. Mol Ecol. 2016;25:1175–91. 10.1111/mec.13464. 26558479

[95] Hart AF, Verbeeck J, Ariza D, Cejas D, Ghisbain G, Honchar H, et al. Signals of adaptation to agricultural stress in the genomes of two European bumblebees. Front Genet. 2022;13:993416. 10.3389/fgene.2022.993416. PMC9579324 36276969

[96] Jones FC, Grabherr MG, Chan YF, Russell P, Mauceli E, Johnson J, et al. The genomic basis of adaptive evolution in threespine sticklebacks. Nature. 2012;484:55–61. 10.1038/nature10944. PMC3322419 22481358

[97] Martinez Barrio A, Lamichhaney S, Fan G, Rafati N, Pettersson M, Zhang H, et al. The genetic basis for ecological adaptation of the Atlantic herring revealed by genome sequencing. Elife. 2016;5:e12081. 10.7554/elife.12081. PMC4854517 27138043

[98] Benjelloun B, Leempoel K, Boyer F, Stucki S, Streeter I, Orozco-terWengel P, et al. Multiple genomic solutions for local adaptation in two closely related species (sheep and goats) facing the same climatic constraints. Mol Ecol. 2024;33:e17257. 10.1111/mec.17257. PMC13084981 38149334

[99] Sang Y, Long Z, Dan X, Feng J, Shi T, Jia C, et al. Genomic insights into local adaptation and future climate-induced vulnerability of a keystone forest tree in East Asia. Nat Commun. 2022;13:6541. 10.1038/s41467-022-34206-8. PMC9626627 36319648

[100] Martín-Blázquez R, Calhoun AC, Sadd BM, Cameron SA. Gene expression in bumble bee larvae differs qualitatively between high and low concentration imidacloprid exposure levels. Sci Rep. 2023;13:9415. 10.1038/s41598-023-36232-y. PMC10256756 37296299

[101] Melicher D, Torson AS, Anderson TJ, Yocum GD, Rinehart JP, Bowsher JH. Immediate transcriptional response to a temperature pulse under a fluctuating thermal regime. Integr Comp Biol. 2019;59:320–37. 10.1093/icb/icz096. PMC6703998 31173075

[102] Seidenath D, Weig AR, Mittereder A, Hillenbrand T, Brüggemann D, Opel T, et al. Diesel exhaust particles alter gut microbiome and gene expression in the bumblebee Bombus terrestris. Ecol Evol. 2023;13:e10180. 10.1002/ece3.10180. PMC10283033 37351478

[103] Barribeau SM, Schmid-Hempel P, Walser J-C, Zoller S, Berchtold M, Schmid-Hempel R, et al. Genetic variation and microbiota in bumble bees cross-infected by different strains of *C. bombi*. PLoS ONE. 2022;17:e0277041. 10.1371/journal.pone.0277041. PMC9704641 36441679

[104] Gao J, Jin S-S, He Y, Luo J-H, Xu C-Q, Wu Y-Y, et al. Physiological Analysis and Transcriptome Analysis of Asian Honey Bee (Apis cerana cerana) in Response to Sublethal Neonicotinoid Imidacloprid. Insects. 2020;11:753. 10.3390/insects11110753. PMC7692690 33153109

[105] Johnston PR, Rolff J. Immune- and wound-dependent differential gene expression in an ancient insect. Dev Comp Immunol. 2013;40:320–4. 10.1016/j.dci.2013.01.012. 23395998

[106] Kim S, Kim K, Lee JH, Han SH, Lee SH. Differential expression of acetylcholinesterase 1 in response to various stress factors in honey bee workers. Sci Rep. 2019;9:10342. 10.1038/s41598-019-46842-0. PMC6637154 31316163

[107] Dickey M, Walsh EM, Shepherd TF, Medina RF, Tarone A, Rangel J. Transcriptomic analysis of the honey bee (*Apis mellifera*) queen brain reveals that gene expression is affected by pesticide exposure during development. PLoS ONE. 2023;18:e0284929. 10.1371/journal.pone.0284929. PMC10138211 37104526

[108] Giacomini JJ, Adler LS, Reading BJ, Irwin RE. Differential bumble bee gene expression associated with pathogen infection and pollen diet. BMC Genomics. 2023;24:157. 10.1186/s12864-023-09143-5. PMC10053769 36991318

[109] Reitmayer CM, Girling RD, Jackson CW, Newman TA. Repeated short-term exposure to diesel exhaust reduces honey bee colony fitness. Environ Pollut. 2022;300:118934. 10.1016/j.envpol.2022.118934. 35114309

[110] Quinlan GM, Hines HM, Grozinger CM. Leveraging transcriptional signatures of diverse stressors for bumble bee conservation. Mol Ecol. 2025;34:e17626. 10.1111/mec.17626. PMC11754722 39670295

[111] Sánchez-Echeverría K, Castellanos I, Mendoza-Cuenca L, Zuria I, Sánchez-Rojas G. Reduced thermal variability in cities and its impact on honey bee thermal tolerance. PeerJ. 2019;7:e7060. 10.7717/peerj.7060. PMC6557256 31211017

[112] Cuvillier-Hot V, Fisogni A, Doublet V, Guillot S, Holl A-C, Leclercq-Dransart J, et al. Urbanization Shifts Immunometabolism in a Common Bumblebee. Ecol Evol. 2024;14:e70743. 10.1002/ece3.70743. PMC11663479 39712035

[113] Goulson D, Whitehorn P, Fowley M. Influence of urbanisation on the prevalence of protozoan parasites of bumblebees. Ecological Entomology. 2012;37:83–9. 10.1111/j.1365-2311.2011.01334.x.

[114] Youngsteadt E, Appler RH, López-Uribe MM, Tarpy DR, Frank SD. Urbanization increases pathogen pressure on feral and managed honey bees. PLoS ONE. 2015;10:e0142031. 10.1371/journal.pone.0142031. PMC4633120 26536606

[115] Eggenberger H, Frey D, Pellissier L, Ghazoul J, Fontana S, Moretti M. Urban bumblebees are smaller and more phenotypically diverse than their rural counterparts. J Anim Ecol. 2019;88:1522–33. 10.1111/1365-2656.13051. 31233621

[116] Lanuza JB, Collado MÁ, Sayol F, Sol D, Bartomeus I. Brain size predicts bees’ tolerance to urban environments. Biol Let. 2023;19:20230296. 10.1098/rsbl.2023.0296. PMC10684341 38016644

[117] Leonard RJ, Pettit TJ, Irga P, McArthur C, Hochuli DF. Acute exposure to urban air pollution impairs olfactory learning and memory in honeybees. Ecotoxicology. 2019;28:1056–62. 10.1007/s10646-019-02081-7. 31512041

[118] Kardum Hjort C, Dudaniec RY, Olsson P, Ekroos J, Smith HG. Landscape and climate-associated selection in the native and widespread bumblebee. Bombus terrestris. Molecular Ecology. 2025;34:e70141. 10.1111/mec.70141. PMC12573718 41065137

[119] Caizergues AE, Le Luyer J, Grégoire A, Szulkin M, Senar J-C, Charmantier A, et al. Epigenetics and the city: Non-parallel DNA methylation modifications across pairs of urban-forest Great tit populations. Evol Appl. 2022;15:149–65. 10.1111/eva.13334. PMC8792475 35126653

[120] Barkan NP, Chevalier M, Pradervand J-N, Guisan A. Alteration of Bumblebee Venom Composition toward Higher Elevation. Toxins. 2020;12:4. 10.3390/toxins12010004. PMC7020474 31861682

[121] Kleijn D, Winfree R, Bartomeus I, Carvalheiro LG, Henry M, Isaacs R, et al. Delivery of crop pollination services is an insufficient argument for wild pollinator conservation. Nat Commun. 2015;6:7414. 10.1038/ncomms8414. PMC4490361 26079893

[122] Franchini P, Fruciano C, Wood TJ, Shastry V, Goulson D, Hughes WOH, et al. Limited introgression from non-native commercial strains and signatures of adaptation in the key pollinator Bombus terrestris. Mol Ecol. 2023;32:5709–23. 10.1111/mec.17151. 37789741

[123] Kardum Hjort C, Paris JR, Olsson P, Herbertsson L, de Miranda JR, Dudaniec RY, et al. Genomic divergence and a lack of recent introgression between commercial and wild bumblebees (Bombus terrestris). Evol Appl. 2022;15:365–82. 10.1111/eva.13346. PMC8965379 35386397

[124] Westrich P. Die Wildbienen Deutschlands. Stuttgart: Verlag Eugen Ulmer; 2018.

[125] Lotterhos KE, Whitlock MC. The relative power of genome scans to detect local adaptation depends on sampling design and statistical method. Mol Ecol. 2015;24:1031–46. 10.1111/mec.13100. 25648189

[126] Foll M, Gaggiotti OE, Daub JT, Vatsiou A, Excoffier L. Widespread Signals of Convergent Adaptation to High Altitude in Asia and America. Am J Hum Genet. 2014;95:394–407. 10.1016/j.ajhg.2014.09.002. PMC4185124 25262650

[127] Hoban S, Kelley JL, Lotterhos KE, Antolin MF, Bradburd G, Lowry DB, et al. Finding the genomic basis of local adaptation: pitfalls, practical solutions, and future directions. Am Nat. 2016;188:379–97. 10.1086/688018. PMC5457800 27622873

[128] Pinheiro J, Bates D, R Core Team. nlme: Linear and Nonlinear Mixed Effects Models. R package version 3.1-164. 2023. Available from: https://CRAN.R-project.org/package=nlme.

[129] Andrews S. FastQC: a quality control tool for high throughput sequence data. Babraham Bioinformatics; 2010. https://www.bioinformatics.babraham.ac.uk/projects/fastqc/.

[130] Sivell O, Sivell D, Smith MN, Natural History Museum Genome Acquisition Lab, Darwin Tree of Life Barcoding collective, Wellcome Sanger Institute Tree of Life Management S and L team. The genome sequence of the large red tailed bumble bee, *Bombus lapidarius* (Linnaeus, 1758). Wellcome Open Res. 2025;10:67. 10.12688/wellcomeopenres.23624.1. PMC11971630 40191528

[131] Darwin Tree of Life Consortium. Bombus lapidarius reference genome assembly iyBomLapi1.1. NCBI Assembly; 2024. Accession GCA_964186655.1. https://www.ncbi.nlm.nih.gov/assembly/GCA_964186655.1.

[132] Darwin Tree of Life Consortium. Bombus terrestris reference genome assembly iyBomTerr1.2. NCBI Assembly; 2022. Accession GCF_910591885.1. https://www.ncbi.nlm.nih.gov/assembly/GCF_910591885.1.

[133] Crowley LM, Sivell O, Sivell D, University of Oxford and Wytham Woods Genome Acquisition Lab, Natural History Museum Genome Acquisition Lab, Darwin Tree of Life Barcoding collective. The genome sequence of the buff-tailed bumblebee, *Bombus terrestris* (Linnaeus, 1758). Wellcome Open Res. 2023;8:161. 10.12688/wellcomeopenres.19248.1. PMC10818103 38283327

[134] Darwin Tree of Life Consortium. Bombus pascuorum reference genome assembly iyBomPasc1.1. NCBI Assembly; 2021. Accession GCA_905332965.1. https://www.ncbi.nlm.nih.gov/assembly/GCA_905332965.1.

[135] Crowley LM, Sivell O, Sivell D, University of Oxford and Wytham Woods Genome Acquisition Lab, Natural History Museum Genome Acquisition Lab, Darwin Tree of Life Barcoding collective. The genome sequence of the common carder bee, *Bombus pascuorum* (Scopoli, 1763). Wellcome Open Res. 2023;8:142. 10.12688/wellcomeopenres.19251.1. PMC10444996 37621574

[136] Vasimuddin M, Misra S, Li H, Aluru S. Efficient architecture-aware acceleration of BWA-MEM for multicore systems. In: 2019 IEEE International Parallel and Distributed Processing Symposium (IPDPS). IEEE; 2019. p. 314–24. 10.1109/IPDPS.2019.00041.

[137] McKenna A, Hanna M, Banks E, Sivachenko A, Cibulskis K, Kernytsky A, et al. The Genome Analysis Toolkit: A MapReduce framework for analyzing next-generation DNA sequencing data. Genome Res. 2010;20:1297–303. 10.1101/gr.107524.110. PMC2928508 20644199

[138] Li H, Handsaker B, Wysoker A, Fennell T, Ruan J, Homer N, et al. The sequence alignment/map format and SAMtools. Bioinformatics. 2009;25:2078–9. 10.1093/bioinformatics/btp352. PMC2723002 19505943

[139] Danecek P, Bonfield JK, Liddle J, Marshall J, Ohan V, Pollard MO, et al. Twelve years of SAMtools and BCFtools. Gigascience. 2021;10:giab008. 10.1093/gigascience/giab008. PMC7931819 33590861

[140] Danecek P, Auton A, Abecasis G, Albers CA, Banks E, DePristo MA, et al. The variant call format and VCFtools. Bioinformatics. 2011;27:2156–8. 10.1093/bioinformatics/btr330. PMC3137218 21653522

[141] Puritz JB, Hollenbeck CM, Gold JR. dDocent: a RADseq, variant-calling pipeline designed for population genomics of non-model organisms. PeerJ. 2014;2:e431. 10.7717/peerj.431. PMC4060032 24949246

[142] Manichaikul A, Mychaleckyj JC, Rich SS, Daly K, Sale M, Chen WM. Robust relationship inference in genome-wide association studies. Bioinformatics. 2010;26:2867–73. 10.1093/bioinformatics/btq559. PMC3025716 20926424

[143] Purcell S, Neale B, Todd-Brown K, Thomas L, Ferreira MAR, Bender D, et al. PLINK: a tool set for whole-genome association and population-based linkage analyses. Am J Hum Genet. 2007;81:559–75. 10.1086/519795. PMC1950838 17701901

[144] Hemstrom W, Grummer JA, Luikart G, Christie MR. Next-generation data filtering in the genomics era. Nat Rev Genet. 2024;25:750–67. 10.1038/s41576-024-00738-6. 38877133

[145] Gruber B, Unmack PJ, Berry OF, Georges A. dartr: An r package to facilitate analysis of SNP data generated from reduced representation genome sequencing. Mol Ecol Resour. 2018;18:691–9. 10.1111/1755-0998.12745. 29266847

[146] Schmidt TL, Jasper M-E, Weeks AR, Hoffmann AA. Unbiased population heterozygosity estimates from genome-wide sequence data. Methods Ecol Evol. 2021;12:1888–98. 10.1111/2041-210x.13659.

[147] Santiago E, Novo I, Pardiñas AF, Saura M, Wang J, Caballero A. Recent Demographic History Inferred by High-Resolution Analysis of Linkage Disequilibrium. Mol Biol Evol. 2020;37:3642–53. 10.1093/molbev/msaa169. 32642779

[148] Liu H, Jia Y, Sun X, Tian D, Hurst LD, Yang S. Direct determination of the mutation rate in the bumblebee reveals evidence for weak recombination-associated mutation and an approximate rate constancy in insects. Mol Biol Evol. 2017;34:119–30. 10.1093/molbev/msw226. PMC5854123 28007973

[149] Corbin LJ, Liu AYH, Bishop SC, Woolliams JA. Estimation of historical effective population size using linkage disequilibria with marker data. J Anim Breed Genet. 2012;129:257–70. 10.1111/j.1439-0388.2012.01003.x. 22775258

[150] Nadachowska-Brzyska K, Konczal M, Babik W. Navigating the temporal continuum of effective population size. Methods Ecol Evol. 2022;13:22–41. 10.1111/2041-210x.13740.

[151] Menard S. Applied logistic regression analysis. SAGE Publications, Inc.; 2002. 10.4135/9781412983433.

[152] Paradis E, Schliep K. Ape 5.0: an environment for modern phylogenetics and evolutionary analyses in R. Bioinformatics. 2019;35:526–8. 10.1093/bioinformatics/bty633. 30016406

[153] Lenth R. emmeans: estimated marginal means, aka least-squares means [R package]. 2025. https://cran.r-project.org/package=emmeans.

[154] Pembleton LW, Cogan NOI, Forster JW. StAMPP: an R package for calculation of genetic differentiation and structure of mixed-ploidy level populations. Mol Ecol Resour. 2013;13:946–52. 10.1111/1755-0998.12129. 23738873

[155] Dray S, Dufour A-B. The ade4 package: implementing the duality diagram for ecologists. J Stat Softw. 2007;22:1–20. 10.18637/jss.v022.i04.

[156] Frichot E, Mathieu F, Trouillon T, Bouchard G, Francois O. Fast and efficient estimation of individual ancestry coefficients. Genetics. 2014;196:973–83. 10.1534/genetics.113.160572. PMC3982712 24496008

[157] Frichot E, François O. LEA: An R package for landscape and ecological association studies. Methods Ecol Evol. 2015;6:925–9. 10.1111/2041-210x.12382.

[158] Jombart T, Ahmed I. adegenet 1.3-1: new tools for the analysis of genome-wide SNP data. Bioinformatics. 2011;27:3070–1. 10.1093/bioinformatics/btr521. PMC3198581 21926124

[159] Browning BL, Zhou Y, Browning SR. A One-Penny Imputed Genome from Next-Generation Reference Panels. The American Journal of Human Genetics. 2018;103:338–48. 10.1016/j.ajhg.2018.07.015. PMC6128308 30100085

[160] Browning BL, Tian X, Zhou Y, Browning SR. Fast two-stage phasing of large-scale sequence data. The American Journal of Human Genetics. 2021;108:1880–90. 10.1016/j.ajhg.2021.08.005. PMC8551421 34478634

[161] Oksanen J, Blanchet FG, Kindt R, Legendre P, Minchin PR, O'Hara RB, et al. vegan: Community Ecology Package [R package]. Version 2.3–1. 2015. https://cran.r-project.org/package=vegan.

[162] Caye K, Jumentier B, Lepeule J, François O. LFMM 2: Fast and Accurate Inference of Gene-Environment Associations in Genome-Wide Studies. Mol Biol Evol. 2019;36:852–60. 10.1093/molbev/msz008. PMC6659841 30657943

[163] Frichot E, Schoville SD, Bouchard G, François O. Testing for associations between loci and environmental gradients using latent factor mixed models. Mol Biol Evol. 2013;30:1687–99. 10.1093/molbev/mst063. PMC3684853 23543094

[164] Barrett RDH, Schluter D. Adaptation from standing genetic variation. Trends Ecol Evol. 2008;23:38–44. 10.1016/j.tree.2007.09.008. 18006185

[165] Szpiech ZA, Hernandez RD. selscan: An Efficient Multithreaded Program to Perform EHH-Based Scans for Positive Selection. Mol Biol Evol. 2014;31:2824–7. 10.1093/molbev/msu211. PMC4166924 25015648

[166] Sabeti PC, Varilly P, Fry B, Lohmueller J, Hostetter E, Cotsapas C, et al. Genome-wide detection and characterization of positive selection in human populations. Nature. 2007;449:913–8. 10.1038/nature06250. PMC2687721 17943131

[167] Charlesworth D, Zhang Y, Bergero R, Graham C, Gardner J, Yong L. Using GC Content to Compare Recombination Patterns on the Sex Chromosomes and Autosomes of the Guppy, Poecilia reticulata, and Its Close Outgroup Species. Mol Biol Evol. 2020;37:3550–62. 10.1093/molbev/msaa187. 32697821

[168] Brůna T, Hoff KJ, Lomsadze A, Stanke M, Borodovsky M. BRAKER2: automatic eukaryotic genome annotation with GeneMark-EP+ and AUGUSTUS supported by a protein database. NAR Genom Bioinform. 2021;3:lqaa108. 10.1093/nargab/lqaa108. PMC7787252 33575650

[169] Brůna T, Li H, Guhlin J, Honsel D, Herbold S, Stanke M, et al. Galba: genome annotation with miniprot and AUGUSTUS. BMC Bioinformatics. 2023;24:327. 10.1186/s12859-023-05449-z. PMC10472564 37653395

[170] nextgenusfs. funannotate: eukaryotic genome annotation pipeline [software]. GitHub. https://github.com/nextgenusfs/funannotate. Accessed 21 Dec 2024.

[171] Haas BJ, Salzberg SL, Zhu W, Pertea M, Allen JE, Orvis J, et al. Automated eukaryotic gene structure annotation using EVidenceModeler and the Program to Assemble Spliced Alignments. Genome Biol. 2008;9:R7. 10.1186/gb-2008-9-1-r7. PMC2395244 18190707

[172] Seppey M, Manni M, Zdobnov EM. BUSCO: assessing genome assembly and annotation completeness. In: Kollmar M, editor. Gene prediction: methods and protocols, vol. 1962. New York, NY: Humana; 2019. p. 227–45. 10.1007/978-1-4939-9173-0_14. 31020564

[173] Lawrence M, Huber W, Pagès H, Aboyoun P, Carlson M, Gentleman R, et al. Software for computing and annotating genomic ranges. PLoS Comput Biol. 2013;9:e1003118. 10.1371/journal.pcbi.1003118. PMC3738458 23950696

[174] Lawrence M, Gentleman R, Carey V. Rtracklayer: an R package for interfacing with genome browsers. Bioinformatics. 2009;25:1841–2. 10.1093/bioinformatics/btp328. PMC2705236 19468054

[175] Quinlan AR, Hall IM. BEDTools: a flexible suite of utilities for comparing genomic features. Bioinformatics. 2010;26:841–2. 10.1093/bioinformatics/btq033. PMC2832824 20110278

[176] Cingolani P, Platts A, Wang LL, Coon M, Nguyen T, Wang L, et al. A program for annotating and predicting the effects of single nucleotide polymorphisms, SnpEff: SNPs in the genome of *Drosophila melanogaster* strain w1118; iso-2; iso-3. Fly. 2012;6:80–92. 10.4161/fly.19695. PMC3679285 22728672

[177] Stolle E, Wilfert L, Schmid-Hempel R, Schmid-Hempel P, Kube M, Reinhardt R, et al. A second generation genetic map of the bumblebee Bombus terrestris (Linnaeus, 1758) reveals slow genome and chromosome evolution in the Apidae. BMC Genomics. 2011;12:48. 10.1186/1471-2164-12-48. PMC3034698 21247459

[178] Alexa A, Rahnenführer J, Lengauer T. Improved scoring of functional groups from gene expression data by decorrelating GO graph structure. Bioinformatics. 2006;22:1600–7. 10.1093/bioinformatics/btl140. 16606683

[179] Alexa A, Rahnenführer J. topGO: enrichment analysis for Gene Ontology [R package]. Version 2.54.0. 2023. 10.18129/b9.bioc.topgo.

[180] Götz S, García-Gómez JM, Terol J, Williams TD, Nagaraj SH, Nueda MJ, et al. High-throughput functional annotation and data mining with the Blast2GO suite. Nucleic Acids Res. 2008;36:3420–35. 10.1093/nar/gkn176. PMC2425479 18445632

[181] Theodorou P. Scripts, variant call format files and Bombus lapidarius genome annotation for “Urbanisation drives parallel genomic divergence across cities and bumble bee species.” 2026. Figshare. 10.6084/m9.figshare.26303974.

[182] Emms DM, Kelly S. OrthoFinder: solving fundamental biases in whole genome comparisons dramatically improves orthogroup inference accuracy. Genome Biol. 2015;16:157. 10.1186/s13059-015-0721-2. PMC4531804 26243257

[183] Yu G. Gene ontology semantic similarity analysis using GOSemSim. In: Kidder BL, editor. Stem cell transcriptional networks: methods and protocols, vol. 2117. New York, NY: Springer US; 2020. p. 207–15. 10.1007/978-1-0716-0301-7_11. 31960380

[184] Yu G, Li F, Qin Y, Bo X, Wu Y, Wang S. GOSemSim: an R package for measuring semantic similarity among GO terms and gene products. Bioinformatics. 2010;26:976–8. 10.1093/bioinformatics/btq064. 20179076

[185] Theodorou P. Bombus sp. raw sequence reads. NCBI BioProject. 2024. Accession PRJNA1117648. https://www.ncbi.nlm.nih.gov/bioproject/PRJNA1117648.

[186] Theodorou P. Parallel urban bumble bee genomics: population genomic analyses of urban adaptation and parallel genomic divergence across bumble bee species. GitHub. 2026. https://github.com/panastheod/Parallel_urban_bumble_bee_genomics.

