# Supplementary material for: Urbanisation drives parallel genomic divergence across cities and bumble bee species
Source: Genome Biol. 2026 Oct 26;27:326. doi: 10.1186/s13059-026-04322-5 (PMC13700247; doi:10.1186/s13059-026-04322-5)
Supplement: Supplementary file 1 [file 13059_2026_4322_MOESM2_ESM.docx]

**Additional file 2**

**Urbanisation drives parallel genomic divergence across cities and bumble bee species**

Panagiotis Theodorou^1,2*^ & Robert J. Paxton^1,2^

^1^ General Zoology, Institute for Biology, Martin Luther University Halle-Wittenberg, Hoher Weg 8, 06120 Halle (Saale), Germany

^2^ German Centre for Integrative Biodiversity Research (iDiv) Halle-Jena-Leipzig, Puschstrasse 4, 04103 Leipzig, Germany

^*^ Correspondence:

Panagiotis Theodorou, General Zoology, Institute for Biology, Martin Luther University Halle-Wittenberg, Hoher Weg 8, 06120 Halle, Germany

Phone: +49 (0)345 55 26511, FAX: +49 (0)345 55 27428, Email: [panatheod@gmail.com](mailto:panatheod@gmail.com)

**Fig. S1.** Contemporary effective population size (Nₑ) trajectories inferred using the linkage disequilibrium-based method GONE for urban and rural populations of *Bombus lapidarius*, *Bombus terrestris*, and *Bombus pascuorum*. Individuals were grouped by ecosystem type (urban versus rural) prior to analysis due to limited sample sizes within individual local populations. Lines represent inferred changes in effective population size across recent generations, plotted on a logarithmic scale. Only for *B. pascuorum* is the inferred population size at urban sites markedly smaller than that at rural sites.

**Fig. S2.** **Relationship between pairwise genetic distance, measured as *F*_ST_/(1 –*F*_ST_) and geographic distance** for **(a)** *Bombus lapidarius*, **(b)** *Bombus terrestris* and **(c)** *Bombus pascuorum*. The relationship between *F*_ST_/(1 –*F*_ST_) and distance was not significant for any of the three species (Mantel tests).

 **Fig. S3. Bumble bee population genetic structure.** sNMF inference of the number of population genetic clusters and d**iscriminant analysis of principal components (DAPC)** scatter plot for **(a)** *Bombus lapidarius*, **(b)** *Bombus terrestris* and **(c)** *Bombus pascuorum* using an LD threshold of --indep 50 5 0.5*.*

# **Fig. S4. Venn diagram of outlier loci** identified by each of the three genotype-environment association (GEA) methods applied (i.e. RDA, LMM and LFMM) for *Bombus lapidarius*, *Bombus terrestris* and *Bombus pascuorum*.

**Fig. S5.** **Principal Component Analysis (PCA) of *B. pascuorum* samples using SNPs on chromosome NC_083489.1**, with points coloured by **(a)** population and **(b)** urban or rural classification for each individual. **(c)** Manhattan plot showing the *P*-values of SNPs across chromosome NC_083489.1 (chromosome 2) in *B. pascuorum*. Spatial correlation can indicate structural variations such as inversions. SNPs highlighted in orange exceed the Bonferroni-corrected significance threshold of 0.01, accounting for multiple testing. Based on asaph, the estimated inversion window spans 14,314,289–20,374,430 bp and contains 25,421 SNPs. Population codes are given in Additional file 1: Table S33.

**Fig. S6.** **Functional enrichment of urban-associated genes.** The central Venn diagram shows overlap among candidate genes identified in *Bombus lapidarius*, *B. terrestris* and *B. pascuorum* based on genotype–environment association analyses (RDA ∩ LMM), together with additional GEA outlier SNPs overlapping XP-nSL selective sweep regions. Bar plots show significantly enriched Gene Ontology (GO) categories for each species grouped by ontology: Biological Process, Molecular Function and Cellular Component. Functional categories include pathways associated with stress responses, neurobiology, signalling, development, metabolism and cellular organisation. The x-axis indicates enrichment significance expressed as -log_10_(P-value) from topology-aware Fisher’s exact tests implemented in topGO, with higher values indicating stronger over-representation relative to the genomic background.

**Fig. S7***.* **Distribution of mean SNP depth across retained loci after filtering in the three bumble bee species.** Histograms show the distribution of mean SNP depth (coverage) for retained SNPs in (a) *Bombus lapidarius*, (b) *Bombus terrestris* and (c) *Bombus pascuorum* following quality filtering. Dashed vertical lines indicate the minimum mean site-depth threshold applied during filtering (--min-meanDP 5). Despite our genotype-level filtering threshold (--minDP 3), retained loci were generally covered substantially above the minimum threshold, with median mean SNP depths around 10× across all species. These results indicate that the filtered datasets were not dominated by marginal low-coverage loci.

**Fig. S8.** Robustness of population structure inference to stricter linkage disequilibrium pruning thresholds. Cross-entropy values from sNMF analyses across alternative numbers of ancestral clusters (K) for (a) *Bombus lapidarius*, (b) *B. terrestris* and (c) *B. pascuorum* using a stricter LD-pruning threshold (--indep 50 5 0.2) than in the manuscript (see Fig. S3). In all three species, cross-entropy profiles remained broadly similar to those obtained under the original pruning threshold (Supplementary Fig. S3) and continued to support weak genome-wide population structure. Also shown is the discriminant analysis of principal components (DAPC) based on the same stricter LD-pruned datasets. Points represent individuals coloured according to sampling region, with filled and open symbols indicating urban and rural populations, respectively. Ellipses represent population clustering. Despite stricter LD pruning than in the manuscript, clustering patterns remained qualitatively similar to the original analyses, for which the LD-pruning threshold was --indep 50 5 0.5, and continued to indicate high connectivity and weak genetic structure across the study region.

**Fig. S9***.* **Subsampling analysis of expected heterozygosity across five populations of *Bombus lapidarius*.** Boxplots show the distribution of unbiased expected heterozygosity (*uH*_EXP_) across 100 random subsamples for each sample size of individuals. Thick coloured lines denote medians. Coloured lines connect median values across sample sizes for each population. *uH*_EXP_ stabilized rapidly, with median values converging to large-sample estimates by approximately n = 4-5 individuals.

**Fig. S10.** **Accuracy of allele frequency estimates across sample sizes.**
Boxplots show the absolute deviation between allele frequencies estimated from subsampled datasets (n = 2 to 11) and those estimated from the full sample (n = 12) across 1,000 randomly selected SNPs. Dashed red lines represent fixed deviation thresholds (0.02, 0.03, and 0.05), and the overlaid labels indicate the percentage of SNPs within each threshold for each subset size. As sample size increases, the deviation decreases, indicating improved accuracy of allele frequency estimation. These results nevertheless demonstrate that relatively small sample sizes can yield allele frequency estimates that closely approximate full-sample values for a large proportion of loci.
